# Marsupial single-cell transcriptomics identifies temporal diversity in mammalian developmental programs

**DOI:** 10.1016/j.devcel.2025.06.037

**Published:** 2025-07-24

**Authors:** Sergio Menchero, Christopher Barrington, Clare Critcher, Wazeer Varsally, Gregorio Alanis-Lobato, Kathy K. Niakan, James M.A. Turner

**Affiliations:** 1 Sex Chromosome Biology Laboratory, The Francis Crick Institute, London NW1 1AT, UK; 2 Bioinformatics & Biostatistics, The Francis Crick Institute, London NW1 1AT, UK; 3 Human Embryo and Stem Cell Laboratory, The Francis Crick Institute, London NW1 1AT, UK; 4 Department of Physiology, Development and Neuroscience, The Centre for Trophoblast Research, University of Cambridge, Cambridge CB2 3EL, UK; 5 Wellcome-MRC Cambridge Stem Cell Institute, University of Cambridge, Cambridge CB2 0AW, UK; 6 Epigenetics Programme, The Babraham Institute, Cambridge CB22 3AT, UK

## Abstract

Single-cell transcriptomics has demonstrated conserved and divergent programs of organogenesis in mammals, but existing studies have focused on eutherians. Marsupials exhibit short gestation and complete development externally, necessitating accelerated differentiation of anterior features required for locomotion and feeding. Hence, they represent a unique outgroup for studying temporal shifts in development, known as heterochrony. Here, we generate a single-cell transcriptomic atlas of gastrulation and early organogenesis in a marsupial, the opossum *Monodelphis domestica*. We identify previously undocumented tissues undergoing heterochrony and find that transcriptional programs that form anterior structures initiate earlier and progress faster relative to eutherians. The result is uncoupling of transcriptional and morphological timelines, revealing unforeseen diversity in mammalian developmental sequences. Using our transcriptomic dataset, we identified translation as a candidate control mechanism by which anterior prioritization is achieved. Our findings provide insight into the asynchronous progression of developmental programs in marsupials.

## Graphical abstract

**Graphical abstract:**
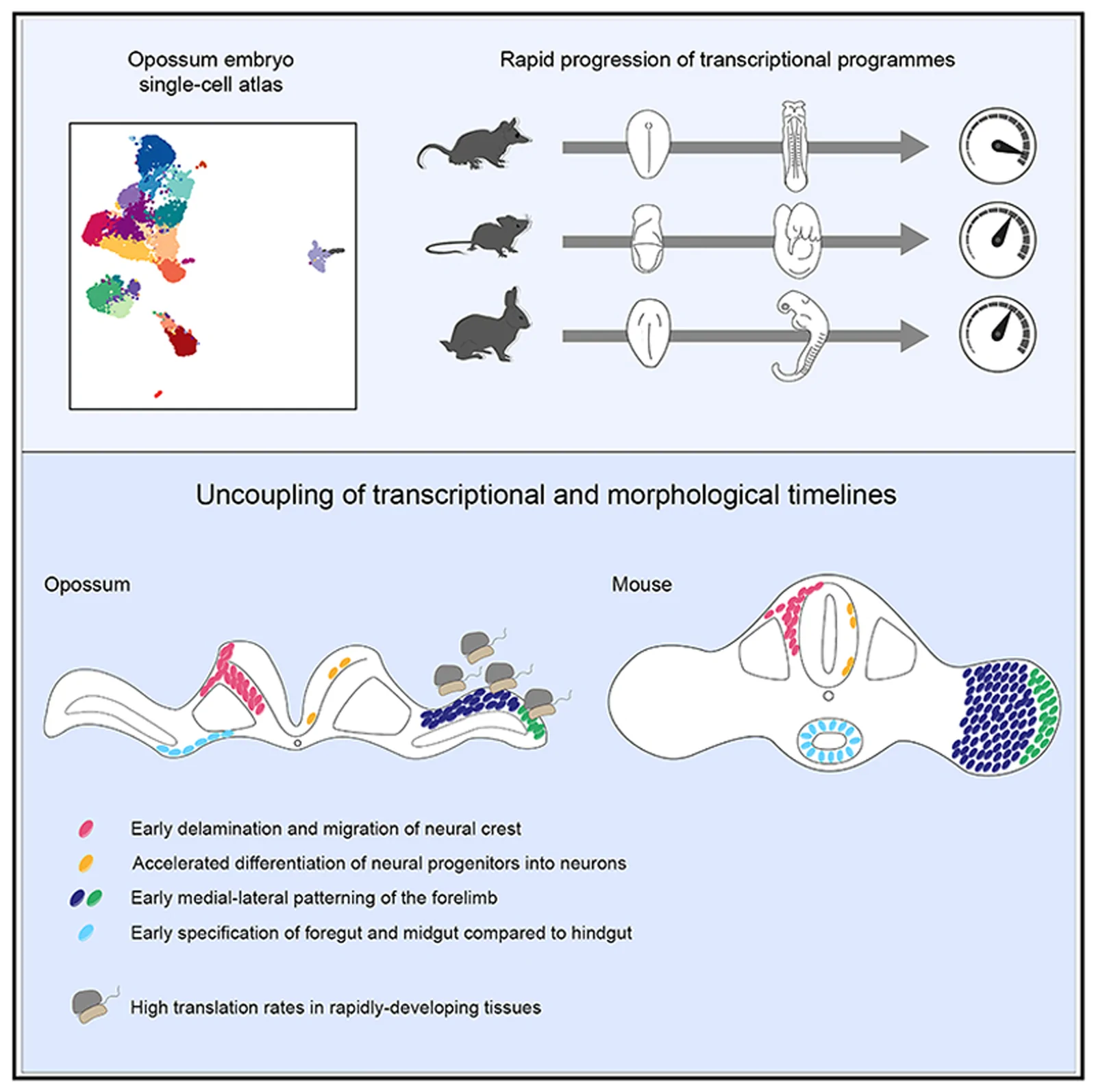

## Introduction

Embryonic development entails the emergence of different cell types driven by the activation of specific genetic and cellular programs. In vertebrates, the gene regulatory networks and morphogenetic changes that drive these transitions are broadly conserved.^1–3^ Nevertheless, in many species, there is a shift in the rate, timing, or order in which certain cell types or tissues arise. This change in timing relative to a related species is known as heterochrony.^4,5^ Developmental heterochrony has been proposed as an evolutionary force that results in phenotypic diversification, with examples across multiple phyla. For instance, heterochrony between the formation of the head and trunk has been linked to the evolution of feeding larvae in bilateria.^6^ An increased rate in the formation of somites results in a higher number of vertebrae typical of snakes.^7^ The delayed and protracted maturation of cortical synapsis is characteristic of the human brain.^8,9^ Despite its implications, the temporal diversification of tissue programs throughout evolution is understudied.

Marsupial (metatherian) neonates exhibit short gestation compared with other mammals, and development is completed externally after birth. As a consequence, they undergo a marked anterior to posterior (A-P, rostral to caudal) gradient in development, with a particularly prominent growth of facial structures and forelimbs.^10^ This A-P heterochrony allows altricial newborns to crawl and receive nutrients from the mother. This phenomenon has been appreciated for decades at the morphological level but less at the molecular level. Existing studies have focused on neural crest and limb formation, where early expression of selected genes has been reported.^11–14^ High-resolution studies could provide a genome-wide landscape of developmental heterochrony, offering deeper insights into the progression of genetic programs in rapidly differentiating tissues. Furthermore, they could identify previously undocumented cases of heterochrony in other tissues and serve as a foundation for exploring potential processes controlling developmental pace.

The expansion of single-cell technologies applied to cells from different tissues, embryonic time points, and species has generated transcriptional atlases of development.^15–24^ Crossspecies comparisons are particularly powerful at unveiling conserved signatures and specific evolutionary shifts. For instance, previous studies have identified conserved core pluripotency factors and divergent primordial germ cell expression programs in mammalian embryos.^25–27^

To gain insight into developmental heterochrony, we have performed single-cell RNA sequencing (scRNA-seq) analysis of gastrulation and early organogenesis in gray short-tailed opossum (*Monodelphis domestica*) embryos and compared this with equivalent eutherian datasets. Our analyses reveal the transcriptomic landscape of neural crest and forelimb specification and identify additional tissues exhibiting early differentiation, such as the spinal cord and the anterior endoderm. Cross-species comparisons and RNA fluorescent *in situ* hybridization chain reaction (HCR RNA-FISH) experiments reveal early specification and rapid progression in the developmental cascade of specific tissues relative to underlying morphological landmarks, such as neural tube closure or limb bud outgrowth. Finally, our analyses identify ribosome biogenesis as a key signature in forelimbs and neural crest, implicating translational regulation as an important mechanism in developmental heterochrony.

## Results

### Cross-species transcriptional equivalence

**Figure 1:**
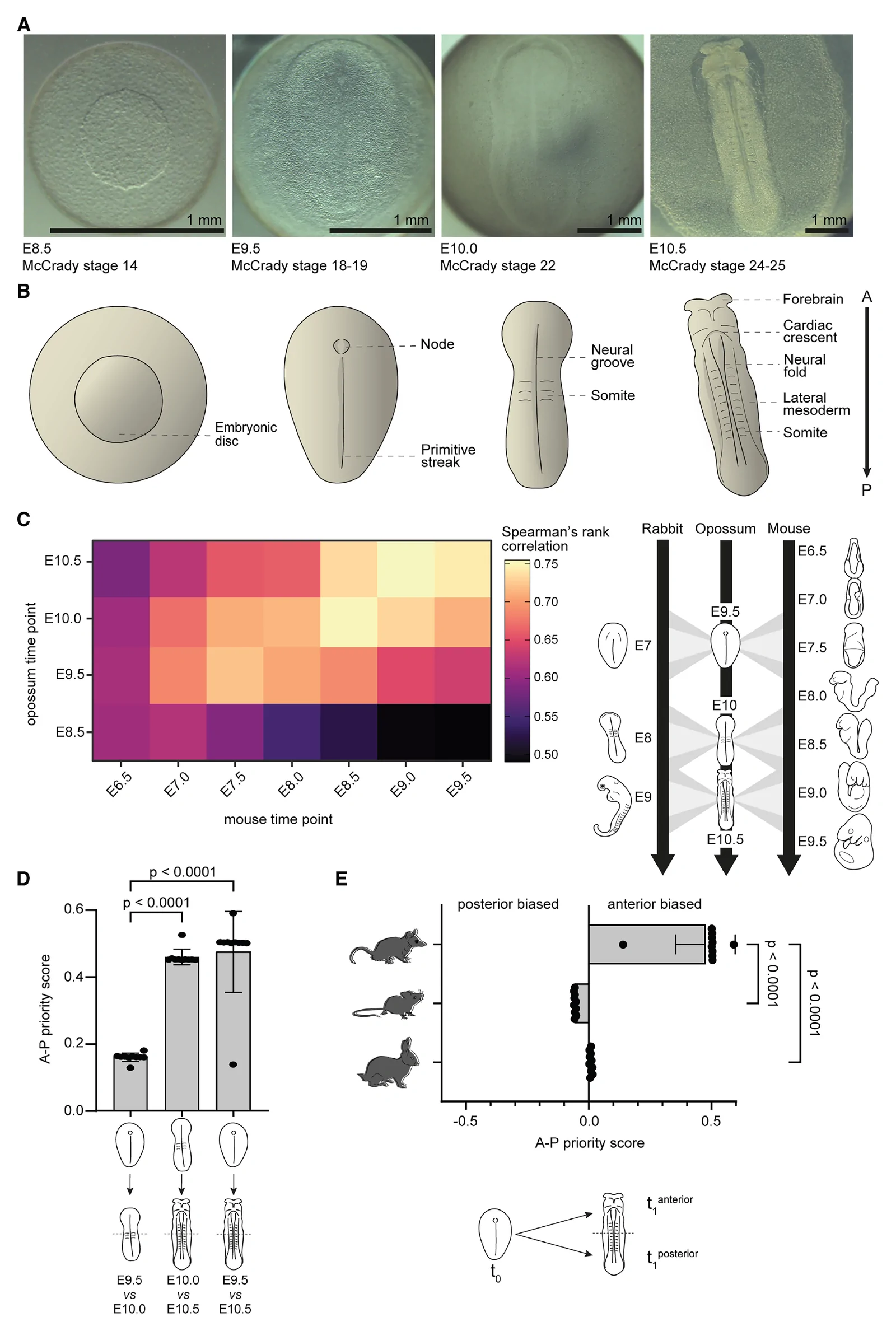
Cross-species transcriptional correspondence (A) Stereo microscope images of opossum embryos at E8.5, E9.5, E10.0, and E10.5. Scale bars, 1 mm. (B) Schematic illustrations of opossum embryos with key features annotated. (C) Heatmap of Spearman’s rank correlation coefficients of gene expression per opossum (vertical) and mouse (horizontal) time point. The intersect of variable features across time points between both species is used to calculate the correlation. Schematic diagrams showing the transcriptional correspondences are summarized on the right panel. See also Figure S2. (D) A-P priority score for opossum embryos using different starting and ending time points (t_0_ = E9.5, t_1_ = E10.0; t_0_ = E10.0, t_1_ = E10.5, and t_0_ = E9.5, t_1_ = E10.5). *p* values after one-way ANOVA with Tukey’s multiple comparison tests are indicated. Data are represented as mean ± SD. (E) A-P priority score for opossum (t_0_ = E9.5; t_1_ = E10.5), mouse (t_0_ = E7.5; t_1_ = E9.0), and rabbit (t_0_ = E7; t_1_ = E9) embryos. *p* values after one-way ANOVA with Tukey’s multiple comparison tests are indicated. Data are represented as mean ± SD.

Gestation in the opossum lasts 14 days, with implantation around days 11–12. Using scRNA-seq, we previously showed that the first week of gestation culminates in the segregation of the three blastocyst lineages: epiblast, trophectoderm, and primitive endoderm.^28^ At embryonic day (E) 11.5, the anterior structures of the opossum embryo, including head and forelimbs, are morphologically advanced^29^ (Figure S1A). We therefore focused on intermediate time points (E8.5, E9.5, E10.0, and E10.5) when gastrulation and early organogenesis take place (Figures 1A and 1B). Several embryos from the same litter were pooled by stage and processed for scRNA-seq using the droplet-based 10X Genomics Chromium platform. To gain spatial resolution, embryos at E10.0 and E10.5 were divided into two parts along the A-P axis. After quality control, 23,669 cells were retained for analysis (Figure S1B; Table S1).

Cross-species comparisons of transcriptional states in embryos can be useful to establish stage equivalences and assess developmental timing.^23,30^ To place our opossum dataset in context, we used available mouse datasets as a reference^17,19^ and addressed developmental correspondence between species. After removing cell clusters associated with extraembryonic tissues (Figure S1C; Table S2), we pseudobulked all cells from each time point. We defined a set of one-to-one orthologs and identified variable features across the time course common to both species to focus on informative genes for developmental progression (Table S3). Opossum E8.5 embryos had a low correlation with all mouse time points (Figure 1C), as they correspond to pre-primitive streak stages. This lower correlation could also represent the hourglass model in which convergence of species’ body plan is maximum at the phylotypic stage but higher diver-sification is found at earlier stages.^18,31^ Opossum E9.5 embryos were most similar to mouse E7.5 embryos, when both species are at the late primitive streak stage (Figure 1C). Interestingly, opossum E10.0 and E10.5 embryos exhibited a transcriptomic profile that in mice is found at a more advanced morphological state. At E10.0, the opossum has 1–2 somites, neurulation has not started, and there is no visible heart, yet the transcriptome resembled that of an E8.5 mouse, in which there are 6–10 somites, the neural tube is starting to close, and the heart is beating (Figure 1C). At E10.5, the opossum is relatively flat, has 10–12 somites, the limb buds are not evident, the neural tube is starting to close, and the heart is beating but not fused in the midline. However, the transcriptome resembled that of an E9.0 mouse, when there are 16–19 somites, the forelimb buds are present, the neural tube is closed, the lens vesicle is forming, the gut tube is closed, and the heart is looping (Figure 1C). We observed a similar phenomenon when comparing opossum with rabbit^20^ and macaque embryos^23^: opossum E10.5 embryos corresponded transcriptionally to rabbit E9 embryos (Figure S1D) and to macaque E26–E29 embryos (Figure S1E). At these stages in rabbit and macaque embryos, the neural tube is closed, the heart is prominent, the optic vesicle is evident and the forelimb bud is emerging.^32,33^ To deconvolve the origin of this similarity, we compared our opossum anterior and posterior datasets individually with these eutherian species. The correlation coefficient was higher for the anterior than the posterior opossum dataset when compared with morphologically advanced eutherian stages (Figure S1F).

To delineate when anterior structures become prioritized over posterior ones during opossum development, we developed an “A-P priority” score by calculating the number of differentially expressed genes in the anterior and posterior datasets compared with an earlier time point (Figure S1G; Table S3). To eliminate the potential impact of having different numbers of cells in anterior and posterior datasets, we performed random downsampling and ten iterations for each calculation. A-P prioritization was evident between E10.0 and E10.5. The A-P score was + 0.16 in the E9.5 – E10.0 comparison but increased to + 0.46 in the E10.0 to E10.5 comparison. Consequently, the A-P score was also higher in the E9.5 to E10.5 comparison (Figure 1D). These results indicate a greater change in gene expression in the anterior than the posterior embryo region from the onset of organogenesis. To contextualize this with eutherians, we used mouse^17^ and rabbit^20^ anterior and posterior datasets. Following the transcriptional equivalence described above, we compared mouse E7.5 with E9.0 and rabbit E7 with E9 anterior and posterior datasets. The anterior bias observed in the opossum was not observed in the mouse (–0.05) or rabbit (+0.01) (Figure 1E). These findings were consistent when analyzing specific tissues that expand along the A-P axis: endoderm, neural ectoderm, and non-neural ectoderm (Figure S1H). These analyses demonstrate uncoupling between the transcriptional and morphological progression of opossum embryos, mainly due to the rapid transcriptional differentiation of the anterior part of the embryo at the onset of organogenesis.

### Conserved developmental progression at gastrulation

**Figure 2:**
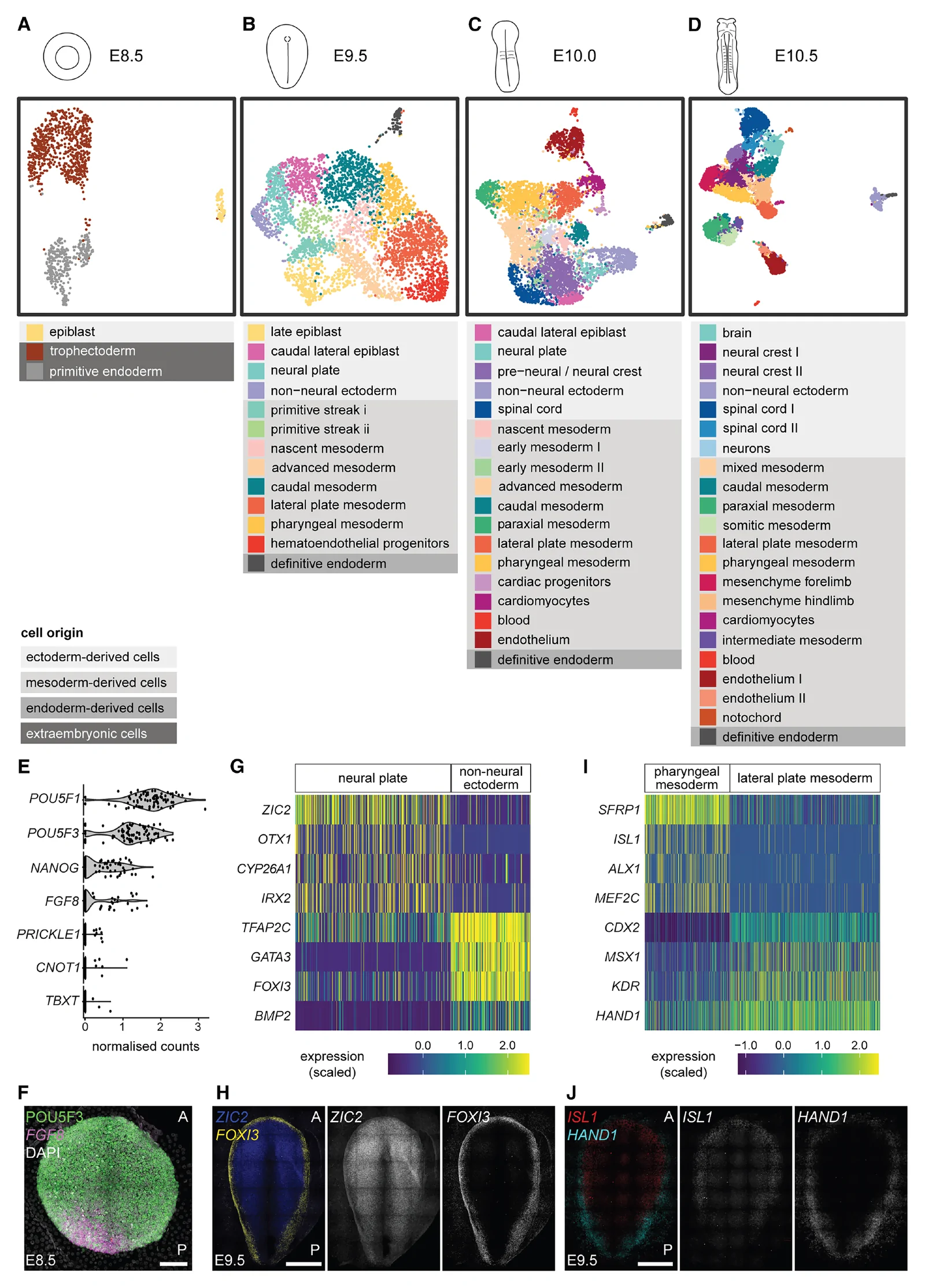
Opossum embryo single-cell clustering and annotation (A–D) UMAP of opossum scRNA-seq data from E8.5, E9.5, E10.0, and E10.5 embryos. Clusters were annotated using module scores. Module scores are based on the combinatorial expression of markers reported in Table S2. (E) Violin plots for the expression of pluripotency (*POU5F1, POU5F3*, and *NANOG*) and pre-primitive streak markers (*FGF8, PRICKLE1, CNOT1*, and *TBXT*) in the epiblast of E8.5 embryos. (F) Expression of POU5F3 (HCR immunofluorescence) and *FGF8* (HCR RNA-FISH) in the epiblast of an E8.5 embryo. Nuclei are stained with DAPI. Image is a maximal projection. A, anterior; P, posterior. Scale bar, 100 μm. (G) Heatmap of genes enriched in neural plate and non-neural ectoderm at E9.5. (H) Expression of *ZIC2* (neural plate) and *FOXI3* (non-neural ectoderm) by HCR RNA-FISH in an E9.5 embryo. Images are maximal projections. A, anterior; P, posterior. Scale bar, 500 μm. (I) Heatmap of genes enriched in pharyngeal mesoderm and lateral plate mesoderm at E9.5. (J) Expression of *ISL1* (pharyngeal mesoderm) and *HAND1* (lateral plate mesoderm) by HCR RNA-FISH in an E9.5 embryo. Images are maximal projections. A, anterior; P, posterior. Scale bar, 500 μm.

Next, we used uniform manifold approximation and projection (UMAP) to visualize clusters of cell types within our single-cell data (Figures 2A–2D). To aid assignment of cell types to each cell cluster, we calculated a gene module score based on the combinatorial expression of conserved key marker genes associated with those cell types/states (Table S2; Figures S2 and S3). At E8.5, we found the same clusters that are present at E7.5^28^: the embryonic epiblast, trophectoderm and primitive endoderm (Figures 2A, S1C, and S2A). Pluripotency-associated markers *POU5F1, POU5F3*, and *NANOG* were detected in most epiblast cells. Expression of genes that in the chicken and mouse precede primitive streak formation, e.g., *FGF8, PRICKLE1, CNOT1*, and *TBXT*, was detected in a few epiblast cells (Figure 2E).^34–37^ Immunofluorescence and fluorescent RNA *in situ* hybridization chain reaction (HCR IF + RNA-FISH) revealed that POU5F3 was expressed throughout the epiblast, while *FGF8* expression was polarized to one side of the embryonic disc (Figure 2F). This indicates that symmetry is broken at the molecular level by E8.5, consistent with a previous study.^38^

Transcriptome comparison of mouse and opossum embryos indicated that the late primitive streak stage (mouse E7.5 and opossum E9.5) was similar between both species (Figure 1C). In the mouse, different subtypes of ectoderm and mesoderm are segregated at this point (E7.5).^19^ In the opossum, our clustering approach also identified distinct ectoderm and mesoderm subtypes at E9.5 (Figure 2B). Neural and non-neural ectoderm were segregated, and the expression of key genes such as *ZIC2* and *OTX1* (neural plate) or *TFAP2C, GATA3*, and *FOXI3* (non-neural ectoderm) was enriched in their corresponding clusters (Figure 2G).^39–43^ Similarly, the most anterior part of the opossum lateral plate mesoderm, the pharyngeal mesoderm, had acquired a specific transcriptional signature (Figure 2B). This included the expression of cardiac progenitor markers such as *MEF2C* and *ISL1*,^44,45^ that distinguished it from the most posterior lateral plate mesoderm (Figure 2I). Using HCR RNA-FISH, we verified that ectoderm and mesoderm subtypes were also spatially segregated as expected (Figures 2H and 2J). Thus, mouse E7.5 and opossum E9.5 embryos exhibit broadly similar cell types.

### Advanced developmental progression at organogenesis

While opossum embryos exhibited a similar profile to the mouse at the late primitive streak stage, cross-species comparisons revealed advanced progression at organogenesis (Figure 1C). At E10.0, we found transcriptional signatures corresponding to some advanced cell types, including paraxial mesoderm, cardiomyocytes, spinal cord, and neural crest (Figure 2C). It was surprising to find cells with a neural crest signature given the flat morphology of the opossum embryo at this point, since *bona fide* neural crest cells are only found in the mouse when the body plan is more advanced, around E8.25–E8.5.^19^

By E10.5, clusters that defined mature cell types became a prominent feature of the dataset. Forelimb and hindlimb domains were segregated, the neural crest and spinal cord clusters were subdivided, and a cluster of neurons was identified (Figure 2D), something that only happens in the mouse when the neural tube has been fully closed around E9.5.^17,46^ Based on the literature in other marsupials,^47^ we expected very few primordial germ cells (PGCs) to be present at these time points. Indeed, we observed a negligible number of cells expressing conserved PGC markers (*DDX4, SSEA1*, and *NANOG*) at both E10 and E10.5 (Figures S4A and S4B).

These findings support the transcriptional equivalence between opossum and mouse embryos using pseudobulk cross-species comparisons (Figure 1C). To confirm whether this equivalence was maintained at the single-cell level, we performed cross-species comparisons in two iconic cell types known to exhibit heterochrony in marsupials: the neural crest and the limbs. Using neural crest cells from mouse and rabbit single-cell datasets,^17,19,20^ we built a reference UMAP for each species. Next, we subsetted and embedded opossum neural crest cells into the reference mouse and rabbit UMAPs (Figures S4C and S4D). Based on the identity of the closest reference cells, opossum cells acquired a predicted mouse and rabbit equivalent time point and cell type (Table S4). Opossum E10.0 neural crest cells were predicted to correspond to mouse E8.5 and E9.0 migratory neural crest cells. By E10.5, a high proportion of opossum cells reached a mouse E9.5 equivalence and branchial arch neural crest identity, consistent with a more differentiated state (Figure S4C). Similarly, when embedded in the rabbit UMAP, opossum E10.0 neural crest cells had a migratory neural crest identity, while a high proportion of E10.5 cells showed a cranial neural crest identity (Figure S4D). We performed the same analysis using lateral plate and limb mesoderm cells (Figures S4E and S4F). Opossum cells were predicted to correspond to mouse E8.5 and E9.0, and rabbit E8 and E9. Opossum lateral plate mesoderm cells, independently of their developmental time point, were associated with earlier mouse and rabbit time points. Opossum forelimb cells occupied a more advanced signature, while hindlimb cells sat closer to the lateral plate mesoderm identity (Figures S4E and S4F), revealing asynchronicities between both limb fields.

These analyses confirm accelerated developmental progression at the onset of organogenesis in the opossum, signifying the first signs of heterochrony. To better understand this rapid progression, we focused on tissues whose early differentiation is important for the survival of the marsupial neonate.

### Early specification and migration of the neural crest

The neural crest is a migratory multipotent cell population that is specified in the ectoderm, at the border between the neural tube and non-neural ectoderm, and gives rise to a wide variety of cell types including bone, cartilage, neurons, glia, and melanocytes.^48^ In vertebrates, neural crest precursors are induced during gastrulation^49,50^ but *bona fide* neural crest cells, characterized by the expression of *SOX9* and *SNAI1*/*2* among others, are only found once neurulation begins.^51^ Subsequently, they upregulate *SOX10*, delaminate, and migrate to their definitive domains. In mammals, delamination of the most-anterior neural crest cells, the cranial neural crest, occurs prior to neural tube closure, while delamination in the trunk and posterior part of the embryo happens after neural tube closure.^52^ Eventually, around E9.5 in the mouse and rabbit, cranial neural crest cells populate the frontonasal prominence and pharyngeal arches, where they acquire an ectomesenchyme fate and contribute to the craniofacial skeleton.^50,53^

**Figure 3:**
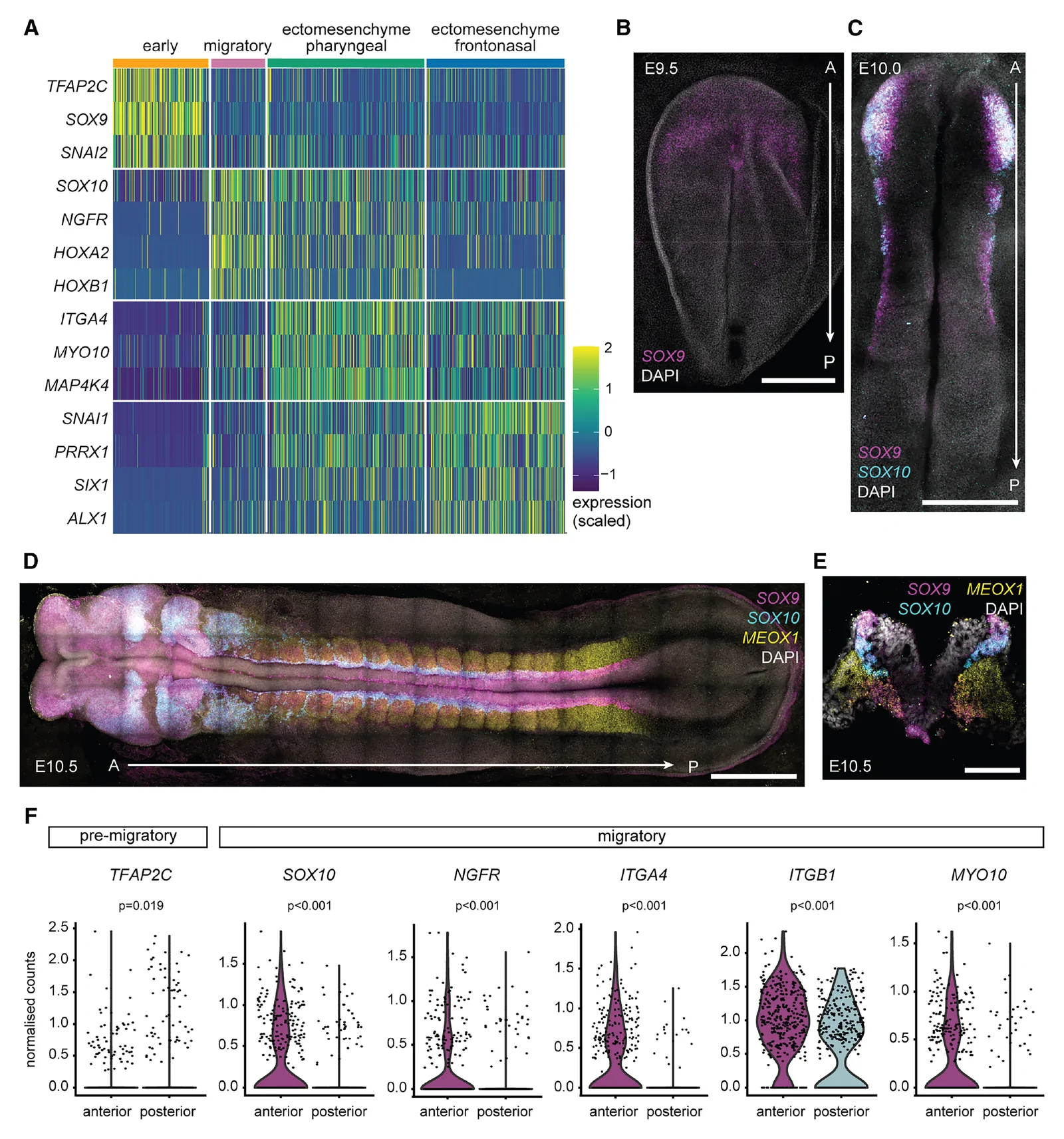
Early specification and migration of the opossum neural crest (A) Heatmap of genes enriched in different clusters of anterior neural crest cells from opossum E10.0 and E10.5 embryos. (B) Expression of *SOX9* by HCR RNA-FISH in an E9.5 embryo. Nuclei are stained with DAPI. Image is a maximal projection. A, anterior; P, posterior. Scale bar, 500 μm. (C) Expression of *SOX9* and *SOX10* by HCR RNA-FISH in an E10.0 embryo. Nuclei are stained with DAPI. Image is a maximal projection. A, anterior; P, =posterior. Scale bar, 500 μm. (D) Expression of *SOX9, SOX10*, and *MEOX1* by HCR RNA-FISH in an E10.5 embryo. Nuclei are stained with DAPI. Image is a maximal projection. A, anterior; P, posterior. Scale bar, 500 μm. (E) Transversal section showing expression of *SOX9, SOX10*, and *MEOX1* by HCR RNA-FISH in an E10.5 embryo. Nuclei are stained with DAPI. Image is a maximal projection. Scale bar, 100 μm. (F) Violin plots showing expression of pre-migratory and migratory neural crest markers in cells from anterior and posterior datasets. *p* values after Student’s t test are shown.

Given that marsupials exhibit rapid development of craniofacial structures, we characterized when the neural crest is specified and when it migrates and differentiates. We identified sub-populations of anterior neural crest using conserved markers of the gene regulatory network^48,54^ and validated our findings using HCR RNA-FISH. Our analysis revealed interesting similarities and differences from eutherians. We identified four opossum neural crest subpopulations and identified differentially expressed genes in each (Figures 3A and S5A). The first, formed mostly at E10.0, was enriched in neural crest specification and delamination markers, including *TFAP2C, SOX9*, and *SNAI2* (Figures 3A and S5B). While this finding suggests that the neural crest is specified at E10.0, HCR-FISH demonstrated that *SOX9* was already expressed in the anterior neural plate at E9.5, and a subset of these cells also expressed TFAP2A/C (Figures 3B and S5C). This is earlier than observed in mouse and chicken, in which *SOX9* is first expressed around the time of somitogenesis,^14^. At E10.0 *SOX9* was confined to the border of the neural plate, consistent with a previous study.^14^ Although *SOX9* was more abundant in the most anterior part, the domain extended to about 2/3 of the embryo length (Figure 3C). A second cluster was enriched in *SOX10* and *NGFR*, genes associated with cell migration (Figure 3A). HCR RNA-FISH confirmed that *SOX10* is expressed from E10.0 but did not extend as posteriorly as *SOX9* (Figure 3C) indicating that, as in eutherians, neural crest differentiation progresses in an A-P manner. At E10.5, anterior neural crest cells had migrated and populated the pharyngeal arches (Figure 3D). However, in contrast to eutherians, trunk neural crest cells had initiated delamination and migration prior to neural tube closure (Figures 3D and 3E). This migration pattern differs from that of mice and rabbits, in which *SOX10* expression starts in five-somite stage embryos, and the population of pharyngeal arches starts in 15-somite embryos.^50,55^

The two remaining neural crest clusters exhibited an enrichment of transcripts linked to an ectomesenchyme fate, such as *SNAI1* and *PRRX1* (Figure 3A). One cluster was enriched in genes associated with late migration (*ITGA4* and *MAP4K4*) and the pharyngeal arch markers *HOXB1* and *HOXA2*, while the other expressed the frontonasal markers *ALX1* and *SIX1* (Figure 3A). To investigate whether differentiation toward other neural crest derivatives had been initiated, and to interrogate differences along the A-P axis, we compared the transcriptome of neural crest cells from E10.5 anterior and posterior datasets (Table S5). Consistent with the previous analysis, anterior neural crest cells exhibited a more advanced migratory state, showing enrichment of genes associated with migration and ectomesenchyme (Figures 3F and S5D). Markers associated with autonomic neural crest such as *ASCL1, HAND2, MSX2*, and *GATA3* were enriched in the posterior population, which encompasses vagal and trunk neural crest (Figure S5D). Genes associated with a sensory fate were not expressed in any neural crest population, indicating that this fate is not yet specified at this time point (Figure S5D). These results suggest that despite the early delamination and migration of both anterior and posterior neural crest, only neural crest cells progressing to an ectomesenchymal fate have acquired a more mature differentiation state at E10.5. We conclude that the prominent differentiation of craniofacial features in marsupials is facilitated by early initiation, early migration and subsequent rapid differentiation of the cranial neural crest.

### Early patterning precedes outgrowth in the forelimb field

Limb formation begins with the specification of the limb fields in the lateral plate mesoderm at specific positions along the A-P axis. The transcription factor *TBX5* determines the forelimb domain, while *PITX1* and its downstream target *TBX4* determine hindlimb identity.^56–60^ These transcription factors activate *FGF10* in the mesoderm, which then activates *FGF8* in the apical ectodermal ridge to create a positive fibroblast growth factor (FGF)-signaling feedback loop that promotes limb outgrowth.^61–63^ Expression of *HOX* genes in the lateral plate mesoderm determines the position of the forelimb, the interlimb flank and the hindlimb.^64^ In most mammals, formation of the fore-limb and hindlimb is asynchronous, with the hindlimb relatively delayed.^65–67^ For instance, in the mouse, the forelimb bud is first evident at E9.0–E9.5, while the hindlimb bud is not until E10.0.^68^

**Figure 4:**
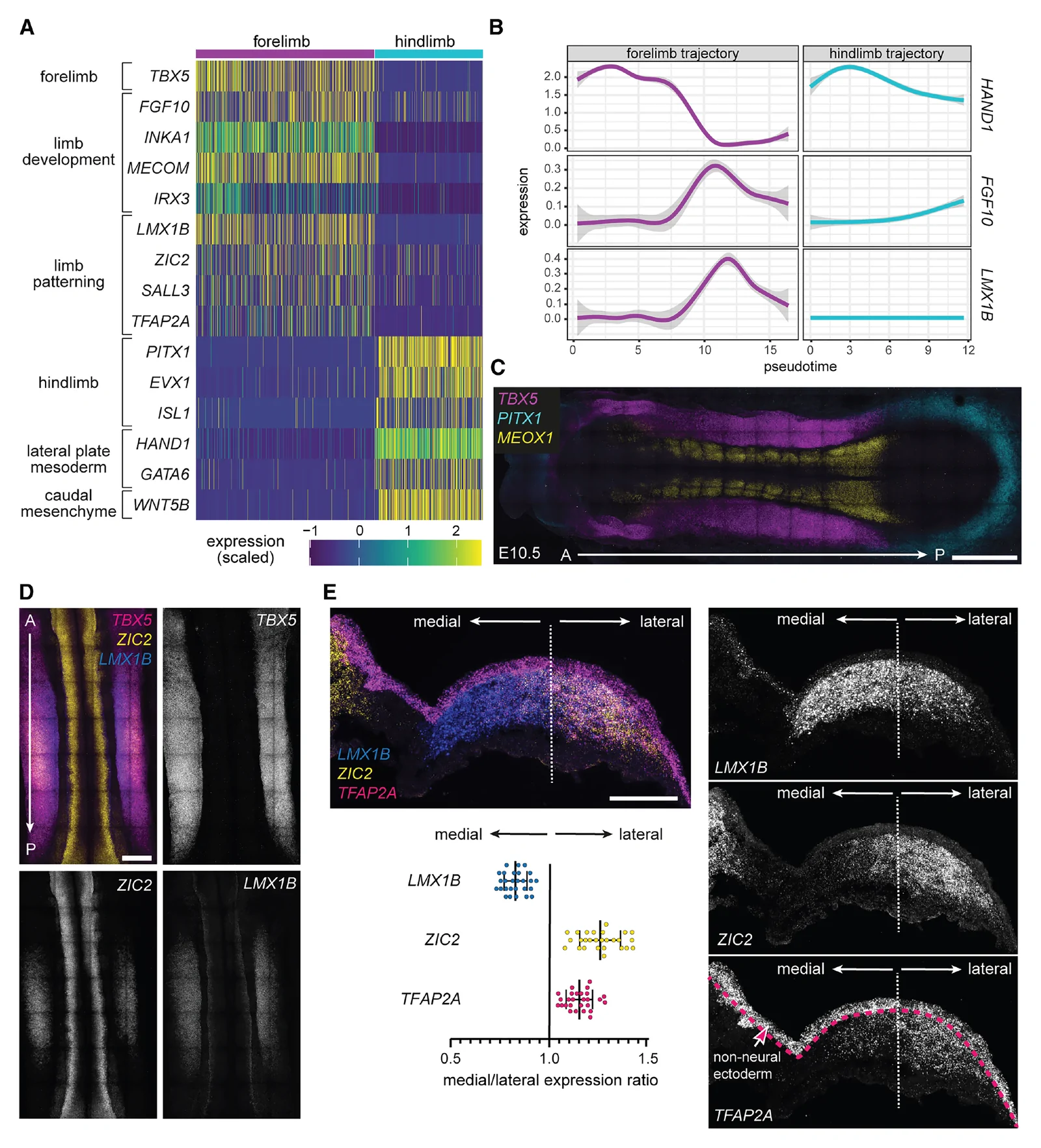
Limb patterning in the opossum embryo (A) Heatmap of genes enriched in the forelimb and hindlimb domains at E10.5. (B) Expression of *HAND1, FGF10*, and *LMX1B* along the forelimb and hindlimb pseudotime trajectory. (C) Expression of *TBX5* (heart and forelimb), *PITX1* (hindlimb), and *MEOX1* (somites) by HCR RNA-FISH in an E10.5 embryo. Image is a maximal projection. A, anterior; P, posterior. Scale bar, 500 μm. (D) Expression of *TBX5, ZIC2*, and *LMX1B* in the forelimb of an E10.5 embryo. Image is a maximal projection. A, anterior; P, posterior. Scale bar, 200 μm. (E) Transversal section showing expression of *LMX1B, ZIC2*, and *TFAP2A* in the opossum E10.5 forelimb by HCR RNA-FISH. *LMX1B* is enriched in the medial domain of the forelimb while *ZIC2* and *TFAP2A* are enriched in the lateral domain. A medial/lateral expression ratio of each gene is plotted (n = 27 sections, 2 embryos). Data are represented as mean ± SD. *TFAP2A* is also expressed in the non-neural ectoderm (magenta dashed line). Images are maximal projections of the sections. Scale bar, 100 μm.

Forelimb-hindlimb heterochrony is extreme in the case of marsupials, which quickly develop their forelimbs to crawl to the teats of the mother after birth.^10^ Based on marker analysis, this heterochrony has been attributed to differences in the timing of limb bud outgrowth, rather than to a delay in hindlimb specification.^12^ Consistent with this model, at E10.5, before any limb bud is obvious, we identified signatures that in other vertebrates typify forelimb and hindlimb identity, including expression of *TBX5* in the forelimb and *PITX1* and *ISL1* in the hindlimb. Nevertheless, transcriptional comparisons revealed that the forelimb and hindlimb were developmentally asynchronous (Figure 4A; Table S5). The forelimb field expressed markers associated with later stages of limb development, including *FGF10, INKA1, MECOM* (also known as *EVI1*), and *IRX3*.^62,69–71^

Conversely, the hindlimb retained expression of the lateral plate mesoderm markers *HAND1* and *GATA6*, although lower than in the lateral plate mesoderm (Figure S5E), and had not yet upregulated *TBX4*. Pseudotime trajectory analysis confirmed this asynchronicity (see STAR Methods for limits of pseudotime application to other lineages). We found two trajectories starting from the lateral plate mesoderm, one extending to the forelimb and the other to the hindlimb cluster (Figures S5F and S5G). Consistent with our initial analysis, *HAND1* expression decreased in the forelimb lineage but was more sustained in the hindlimb. Conversely, *FGF10* and *LMX1B* expression were elevated in the forelimb relative to the hindlimb (Figure 4B).

We used HCR RNA-FISH for *TBX5* and *PITX1* to locate the opossum forelimb and hindlimb domains, respectively. Both genes are initially expressed in broader domains than the future forelimb and hindlimb-forming regions,^12,58,72^ but they are useful markers to distinguish both limb fields. At E10.5, *TBX5* was detected in the cardiac crescent and presumptive forelimb domain, while *PITX1* was expressed in the posterior lateral plate mesoderm, marking the presumptive hindlimb domain (Figure 4C). As previously reported, the opossum *TBX5* domain was more expanded than in other vertebrates,^12^ extending posteriorly to the level of the presomitic mesoderm and abutting the *PITX1* domain. As a consequence, and unlike in chicken and mouse,^57,59^ no interlimb region was observed at E10.5. Therefore, forelimb development is characterized by both heterochrony and heterotopy, i.e., a shift in the spatial patterning of a developmental feature.

Our analysis also revealed expression of limb-patterning genes in the forelimb field (Figure 4A). In mouse and chicken, transcription factors associated with dorsal-ventral (D-V) or proximal-distal patterning (e.g., *LMX1B, ZIC2, SALL3*, and *TFAP2A*^73–77^) are expressed after the limb bud is formed. Surprisingly, these markers were detected in the E10.5 opossum forelimb prior to limb bud elongation. Using HCR RNA-FISH, we visualized local patterning in the forelimb domain at this stage. First, expression of *LMX1B, ZIC2*, and *TFAP2C* was restricted to the middle part of the *TBX5* domain, presumably anticipating the forelimb-forming territory (Figures 4D and S5H). This region coincided spatially with the expression of *FGF8* in the nascent apical ectodermal ridge (Figure S5I). In other vertebrates, restriction of *TBX5* to the definitive forelimb domain precedes expression of *LMX1B, ZIC2*, and *TFAP2C*. The regionalization of these transcripts before *TBX5* in the opossum indicates that different factors are in place to demarcate the limb-forming territory. Second, we observed a medial-lateral patterning, with *LMX1B* enriched in the medial area, while *ZIC2* and *TFAP2A* were segregated to the lateral part of the forelimb (Figure 4E). These results indicate not only that the forelimb and hindlimb domains are specified early but also that the fore-limb undergoes patterning before limb bud outgrowth.

### D-V patterning and first neurons in the spinal cord

Next, we investigated whether our transcriptome dataset would identify heterochrony in other understudied tissues. We focused on tissues whose early differentiation could be an advantage for the marsupial embryo. The spinal cord forms the posterior region of the central nervous system and is the source of neurons that process sensory information and coordinate motor activity. During development, the spinal cord undergoes D-V patterning, induced by opposing morphogen gradients originating from the adjacent non-neural ectoderm and the notochord.^78–80^ These gradients induce expression of transcription factors including *MSX1*/*2, PAX3*, and *NKX6-1* within restricted domains along the D-V axis, leading to specification of distinct neural progenitors.^81^ These progenitors then differentiate into specialized post-mitotic neurons. In mouse, D-V patterning of the spinal cord begins before neural tube closure. However, the differentiation of neural progenitors to neurons only occurs after neural tube closure, around E9.0–E9.5 in the mouse.

**Figure 5:**
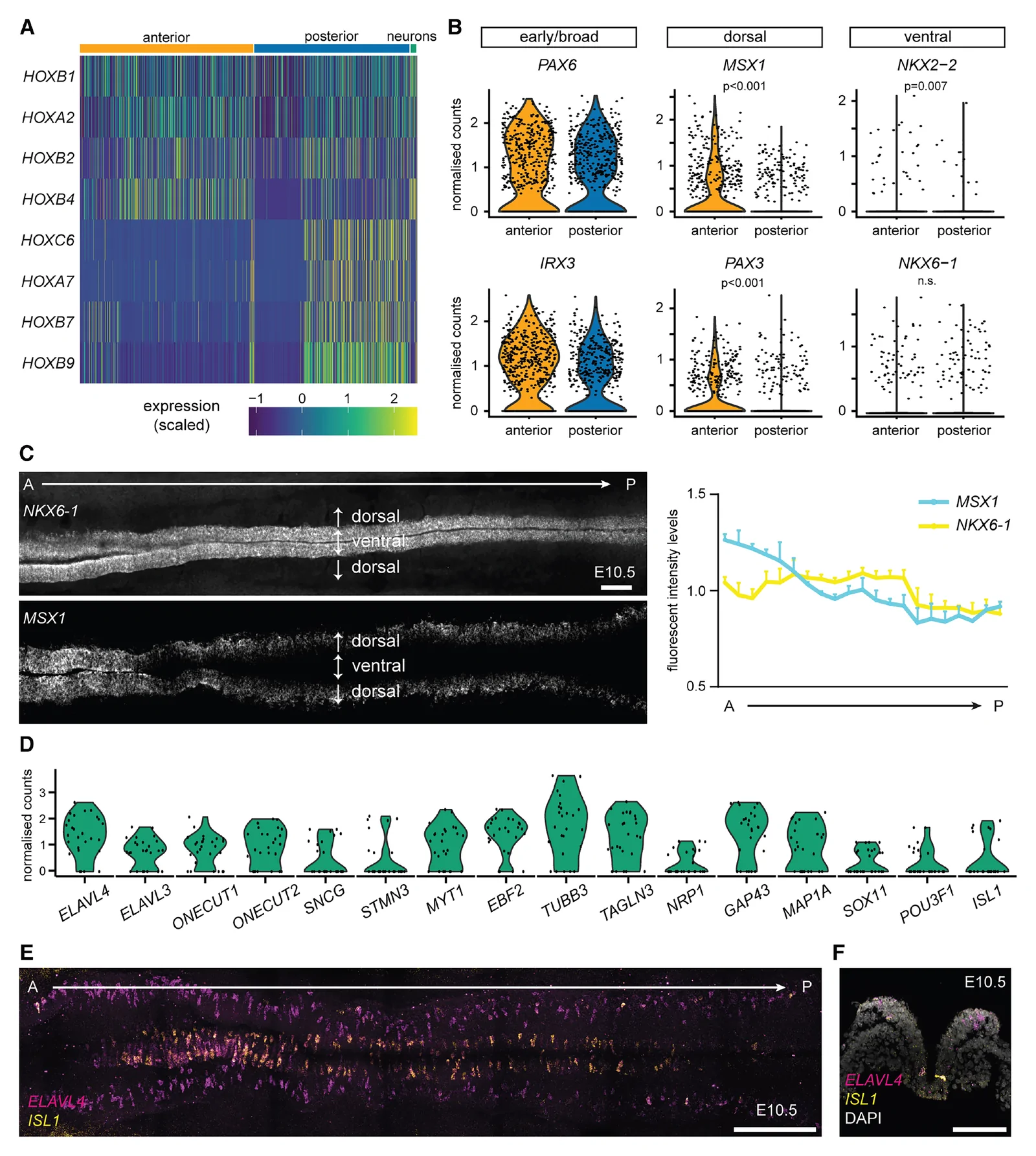
Patterning of the opossum spinal cord (A) Heatmap of *HOX* gene expression in spinal cord and neuron clusters from opossum E10.0 and E10.5 embryos. (B) Violin plots for the expression of early/broad (*PAX6* and *IRX3*), dorsal (*MSX1* and *PAX3*) and ventral (*NKX2-2* and *NKX6-1*) spinal cord markers in anterior and posterior populations. *p* values after Student’s t test are shown. n.s.= not significant. (C) Expression of *NKX6-1* and *MSX1* along the opossum spinal cord. A, anterior; P, posterior. Scale bar, 100 μm. Intensity levels of both markers along the A-P axis were quantified and plotted on the right (21 regions, left and right sides, 2 embryos). Colored line represents the mean expression; error bars represent the standard error of the mean. (D) Violin plots for the expression of neuron-associated markers in opossum neurons at E10.5. (E) Expression of neuronal markers *ELAVL4* and *ISL1* by HCR RNA-FISH in the spinal cord of an E10.5 embryo. Image is a maximal projection. A, anterior; P, posterior. Cells in the midline represent ventral positions. Scale bar, 200 μm. (F) Transversal section showing expression of *ELAVL4* and *ISL1* by HCR RNA-FISH in the spinal cord of an E10.5 embryo. Nuclei are stained with DAPI. Image is a maximal projection. Scale bar, 100 μm.

We investigated whether D-V patterning of the spinal cord was evident in our opossum dataset and found similarities and differences from eutherians. Analysis of the integrated per time point datasets identified one spinal cord cluster at E10, and two spinal cord and one neuron clusters at E10.5 (Figures 2C and 2D). We extracted these cells and re-clustered them agnostic to gestational age, identifying two spinal cord clusters and one neuron cluster (Figures S6A and S6B). The two spinal cord clusters exhibited differences in *HOX* gene expression, indicating that they represented anterior and posterior spinal cord populations.^82^ The presumptive anterior cluster was enriched for *HOXB4* expression, which is characteristic of the anterior cervical region, while the presumptive posterior cluster was enriched for *HOXC6, HOXA7, HOXB7*, and *HOXB9*, which mark the posterior cervical and thoracic regions of the spinal cord (Figure 5A).^12,83^

We then examined whether genes that define distinct domains in the D-V axis were differentially expressed in the anterior and posterior spinal cord clusters.^84,85^ Both clusters expressed dorsal (*MSX1* and *PAX3*) and ventral (*NKX2-2* and *NKX6-1*) markers, although a higher number of cells exhibited a dorsal signature in the anterior population (Figures S6C and S6D). After downsampling to equalize cell numbers, we compared the expression levels of these markers in spinal cord cells from the anterior and posterior datasets, confirming a particular enrichment of dorsal markers in the cervical spinal cord (Figure 5B). HCR RNA-FISH for *NKX6-1* and *MSX1* confirmed the restricted expression of these markers to the ventral and dorsal domains, respectively (Figure 5C). Signal quantification along the A-P axis showed that *MSX1* levels progressively decrease along the A-P axis relative to *NKX6-1* levels. Transverse sections along the A-P axis showed that the *NKX6-1* ventral expression domain remains relatively constant along the A-P axis, while the *MSX1* dorsal expression domain is reduced in posterior sections (Figure S6E). We conclude that, similarly to eutherians, cells respond to the opposing morphogen gradient prior to neural tube closure and the resulting patterning advances in an (A-P) manner.

We then investigated the transcriptional signature of the neuron cluster. We identified an underrepresentation of S and G2M cell-cycle-associated markers (Figure S6F) and observed expression of known post-mitotic pan-neuronal markers, including *ELAVL3*/*4, ONECUT1*/*2, TUBB3, MYT1*, and *GAP43* (Figure 5D).^84,85^ These neurons lacked neural crest-derived neuron markers, confirming that they derive from the central nervous system (Figure S6G). In addition, *ISL1*, a marker of motor neurons and a dorsal interneuron population, was expressed in this cluster. HCR RNA-FISH confirmed that *ISL1*-positive cells were particularly enriched ventrally, in the presumptive motor neuron domain (Figures 5E and 5F). This finding was surprising because, in mouse and chicken, *ISL1* expression is detected after the spinal cord is closed and the number of neural progenitors is higher.^86,87^ Furthermore, we observed a higher density of neurons in the anterior part of the spinal cord, consistent with most neurons expressing *HOXB4* (Figures 5A and 5E). These findings reveal that neural progenitor differentiation in the opossum spinal cord is accelerated, producing post-mitotic neurons, including motor neurons, before neural tube closure.

### Preferential patterning of anterior vs posterior endoderm

Given that at birth marsupials require a respiratory and digestive system, we investigated endoderm differentiation. In the mouse, during gastrulation, definitive endoderm cells intercalate with the underlying extraembryonic endoderm cells to form a layer of cells in the most ventral part of the embryo.^88,89^ This sheet of cells then folds to give rise to an internal primitive gut tube that becomes regionalized along the A-P axis into foregut, midgut, and hindgut domains. These broad domains become gradually specialized in subdomains, and organ buds emerge, giving rise to a variety of tissues including the thyroid, lung, liver, pancreas, and colon.

**Figure 6:**
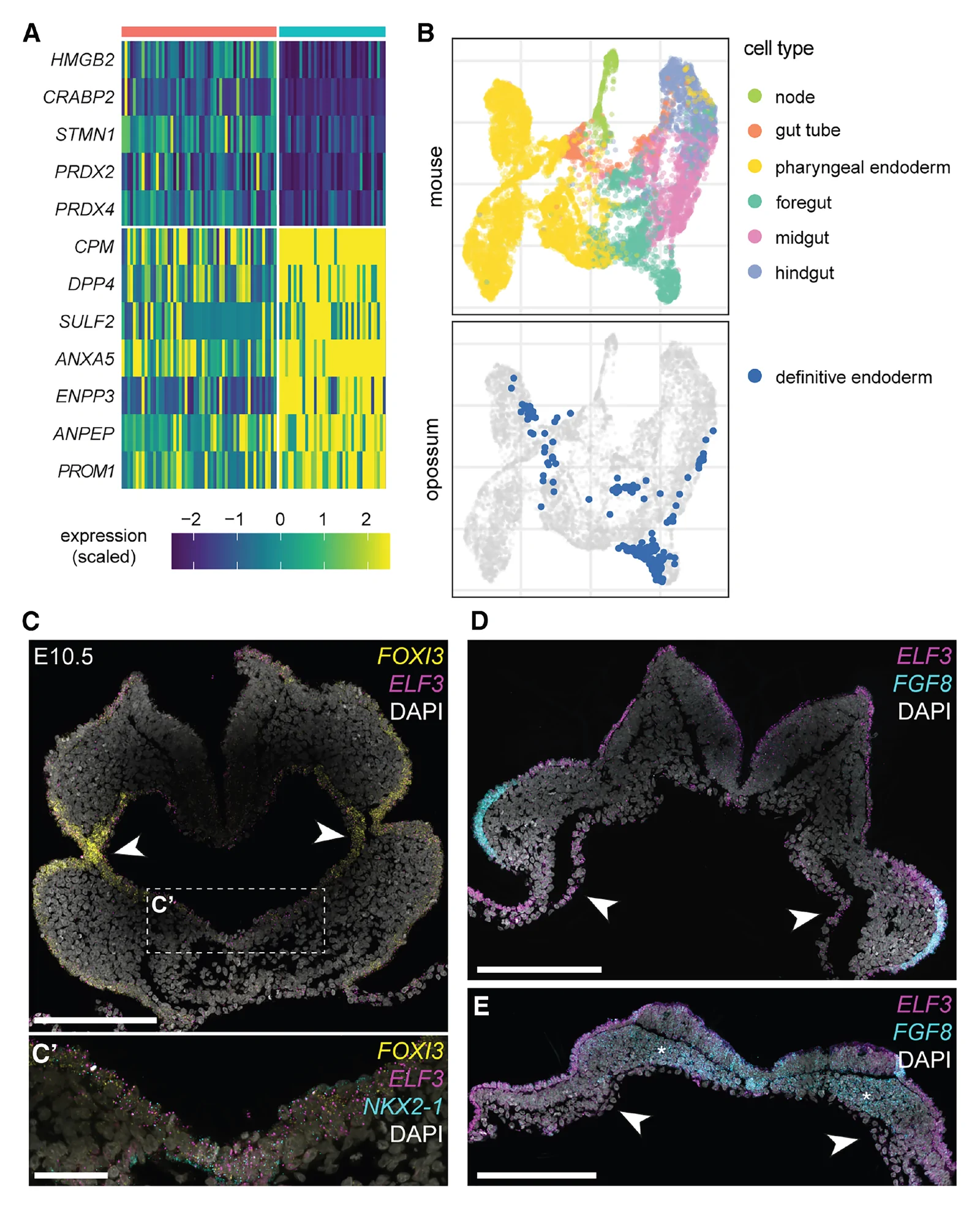
Definitive endoderm differentiation in the opossum (A) Heatmap of genes enriched in two clusters of definitive endoderm cells at E10.5. (B) Mouse reference UMAP of definitive endoderm cells colored by annotated cell type. Embedded opossum definitive endoderm cells are shown in the lower panel. (C) Expression of *FOXI3* and *ELF3* by HCR RNA-FISH in the anterior part of an E10.5 opossum embryo. Image is a maximal projection of a cross section. Arrowheads indicate endoderm cells. *FOXI3* also labels non-neural ectoderm cells. Scale bar, 200 μm. (C^′^) Expression of *FOXI3, ELF3*, and *NKX2-1* in the ventral endoderm cells. Scale bar, 50 μm. (D) Expression of *ELF3* and *FGF8* by HCR RNA-FISH in the trunk of an E10.5 opossum embryo. Image is a maximal projection of a cross section. *ELF3* is expressed in endoderm cells (arrowheads) and non-neural ectoderm cells. *FGF8* is expressed in limb ectoderm. Scale bar, 200 μm. (E) Expression of *ELF3* and *FGF8* by HCR RNA-FISH in the posterior part of an E10.5 opossum embryo. Image is a maximal projection of a cross section. *ELF3* is expressed in non-neural ectoderm cells. *FGF8* is expressed in presomitic mesoderm (*) but absent in endoderm (arrow-heads). Scale bar, 200 μm.

Transcriptional analysis of the E10.5 endoderm revealed two clusters with differential A-P signatures (Figure 6A; Table S5). The first was enriched in anterior foregut markers associated with thymus, thyroid, lung, and liver progenitors, including *HMGB2, CRABP2*, and *STMN1*. The second was enriched in posterior foregut and midgut markers associated with pancreas and small intestine progenitors, such as *CPM, DPP4*, and *ANXA5*.^90^ Notably, we did not detect a distinct hindgut signature. To corroborate this, we embedded opossum definitive endoderm single cells into a mouse reference UMAP^17,19^ (Figure 6B). Based on the coordinates where opossum cells were embedded, they were predicted to have a general gut tube, pharyngeal endoderm (the most anterior part of the foregut), foregut, and midgut signatures. Consistently, no cells exhibited a hindgut signature. We then identified markers using the mouse cell identities and analyzed their expression in opossum definitive endoderm cells. In agreement with the previous analysis, most cells expressed early broad gut tube markers such as *SPINK1* and *TAGLN* (Figure S6H). A high number of cells were also positive for pharyngeal, foregut or midgut markers, but very few cells expressed hindgut markers (Figure S6H).

Using HCR RNA-FISH, we aimed to locate the three gut domains in E10.5 embryos. We selected *FOXI3, ELF3*, and *FGF8* as markers of foregut, midgut, and hindgut, respectively. *FOXI3* was expressed in the lateral region of the pharyngeal endoderm (Figure 6C, arrowheads) while *ELF3* was particularly enriched in the endoderm at the level of the forelimbs (Figure 6D, arrowheads). However, very few posterior endoderm cells expressed *FGF8*, consistent with the hindgut signature being delayed relative to the foregut and midgut (Figure 6E, arrow-heads). The asynchronicity in the gut specification along its A-P axis was further emphasized by the expression of the lung and thyroid progenitor *NKX2-1* on the ventral side of the foregut (Figures 6C′ and S6I). In the mouse, *NKX2-1* expression begins once the gut tube is fully closed, around E9.0,^91^ while the opossum gut tube is only closed in the most anterior region at E10.5. These analyses reveal differences in the timing of endoderm patterning in the opossum.

### Anterior prioritized tissues are marked by higher translation levels

**Figure 7:**
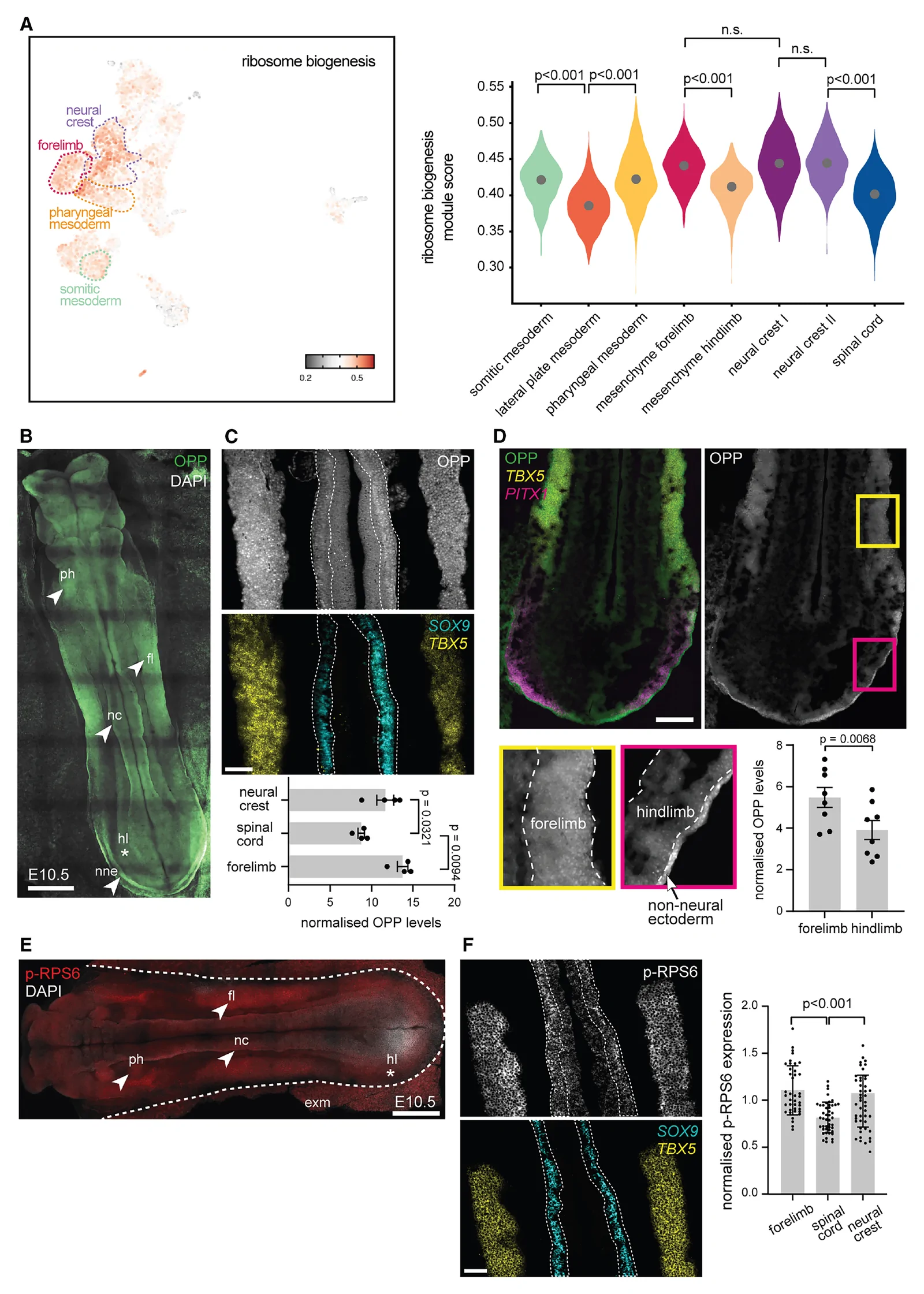
Forelimb and neural crest cells exhibit high protein synthesis and mTOR activity levels (A) Feature plot of opossum E10.5 dataset showing enrichment of module score for ribosome biogenesis. Module score is based on the combinatorial expression of genes reported in Table S6. Violin plots of the same module score in cells of selected clusters are shown on the right panel. *p* values after one-way ANOVA with Tukey’s multiple comparison tests. (B) OPP labeling in E10.5 opossum. Sum slice projection is shown. Nuclei stained with DAPI. White arrowheads indicate areas with high OPP levels: pharyngeal arch (ph), forelimb (fl), neural crest (nc), and non-neural ectoderm (nne). Asterisk indicates hindlimb (hl), which has low OPP levels. Scale bar, 500 μm. (C) Expression of *SOX9* (neural crest) and *TBX5* (forelimb) by HCR RNA-FISH in E10.5 opossum labeled with OPP. A single optical section is shown. Dashed lines indicate the neural crest domain identified by *SOX9-positive* cells. Scale bar, 100 μm. Boxplot showing OPP levels in forelimb (*TBX5* positive), neural crest (*SOX9* positive) and spinal cord regions normalized by cell density (counted nuclei). n = 4 regions, 2 embryos. *p* values after RM one-way ANOVA with Fisher’s LSD multiple comparison tests are indicated. Data are represented as mean ± SD. (D) Expression of *TBX5* (forelimb) and *PITX1* (hindlimb) by HCR RNA-FISH in E10.5 opossum labeled with OPP. The image represents a longitudinal section. Scale bar, 200 μm. Zoom-in regions of the forelimb and hindlimb fields are shown. Note the higher OPP staining in the surrounding non-neural ectoderm. Boxplot showing OPP levels in forelimb (*TBX5* positive) and hindlimb (*PITX1* positive) domains normalized by cell density (counted nuclei). n = 8 sections from 2 embryos. *p* value after paired t test. Data are represented as mean ± SD. (E) p-RPS6 expression by immunofluorescence assay in E10.5 opossum. The sum slice projection is shown. Nuclei stained with DAPI. White arrowheads indicate areas with high p-RPS6 levels: pharyngeal arch (ph), forelimb (fl), and neural crest (nc). Asterisk indicates hindlimb (hl), which has low p-RPS6 levels. A dashed line separates the embryo from the extraembryonic membranes (exm). Scale bar, 500 μm. (F) Expression of p-RPS6 by immunofluorescence and *SOX9* (neural crest) and *TBX5* (forelimb) by HCR RNA-FISH in E10.5 opossum. A single optical section is shown. Dashed lines indicate the neural crest domain identified by *SOX9-positive* cells. Scale bar, 100 μm. Boxplot showing p-RPS6 levels measured along the A-P axis in forelimb (*TBX5* positive), neural crest (*SOX9* positive), and spinal cord regions. n = 46 measurements (3 embryos). *p* values after one-way ANOVA with Tukey’s multiple comparison tests are indicated. Data are represented as mean ± SD.

We investigated whether our scRNA-seq data could be leveraged to identify candidate mechanisms promoting accelerated anterior development. We initially compared the transcriptome of forelimb and hindlimb, which are related tissues exhibiting distinct developmental rates (Table S5). Gene set enrichment analysis (GSEA) revealed that, in addition to forelimb morphogenesis, gene ontology terms related to rRNA biology and ribosome biogenesis were enriched in the forelimb relative to the hindlimb (Figure S7A). Using a gene module score approach (Table S6), we found that ribosome biogenesis-associated genes were also enriched in the neural crest, pharyngeal mesoderm, and somitic mesoderm (Figure 7A). The high rate of somitogenesis at the cervical and thoracic levels^92^ could explain the high ribosome biogenesis levels in somitic mesoderm.

Ribosome biogenesis determines the number of ribosomes in a cell, which dictates its translation capacity.^93^ To test whether tissues enriched in ribosome biogenesis exhibited higher translation rates, we visualized protein synthesis by culturing E10.5 opossum embryos with O-propargyl-puromycin (OPP), which labels nascent polypeptide chains.^94,95^ We observed enrichment of OPP in the forelimb field, pharyngeal arches and neural crest (Figure 7B). OPP labeling in combination with HCR RNA-FISH confirmed enrichment of OPP levels in the neural crest (*SOX9*-positive) and forelimb field (*TBX5*-positive) in comparison with the hindlimb field (*PITX1*-positive) (Figures 7C, 7D, and S7B). The non-neural ectoderm, as the most exposed tissue during incubation, also incorporated high OPP levels. Using HCR RNA-FISH, we confirmed that the posterior region with high OPP levels was the non-neural ectoderm (*TFAP2A*-positive) and not the hindlimb field (Figures S7C and S7D). To delineate at which stage the neural crest and forelimb cells exhibited higher translation, we performed OPP incorporation assays at earlier time points. The anterior neural plate, the precursor of the neural crest, showed no OPP enrichment at E9.5. Subsequently, neural crest cells exhibited OPP enrichment at E10 (Figures S7E–S7G). The lateral plate mesoderm (*HAND1*-positive), the precursor of the forelimb, did not exhibit OPP enrichment at E10 (Figures S7F and S7G). Therefore, increased translation in neural crest and forelimbs occurs at different times, once they acquire their specific identities.

A prominent mechanism regulating translation is the target of rapamycin (mTOR) pathway through the mTOR complex 1 (mTORC1).^96^ Activation of the mTORC1 leads to phosphorylation of translational regulators, including the ribosomal protein S6 (RPS6), to promote protein synthesis. To investigate whether mTOR was preferentially activated in the forelimb and neural crest in the opossum, we performed immunofluorescence for serine-240/244-phosphorylated RPS6 (p-RPS6). p-RPS6 levels were enriched in the forelimb and neural crest cells in the E10.5 opossum (Figures 7E and 7F). Concomitant with OPP incorporation dynamics, p-RPS6 enrichment was also evident in the neural crest (*SOX10* positive) at E10 (Figure S7H). This suggests that the increase in translation observed in rapidly developing tissues may be regulated by mTORC1.

## Discussion

The short gestation of marsupials has favored the evolution of mechanisms prioritizing the differentiation of tissues relevant for the immediate survival of neonates. In this work, we used the opossum as a model to provide insight into developmental heterochrony and the onset of tissue-specific prioritization. Our findings reveal that this bias begins during early organogenesis, with rapid progression of tissues associated with craniofacial structures, locomotor activity, and respiratory and digestive systems. We also identify tissues for which heterochrony has not been previously documented and show how scRNA-seq datasets can be used to identify potential mechanisms regulating developmental timing.

The asynchronous development of anterior and posterior structures in marsupials has been appreciated for decades. Initially, the focus of this uncoupling was on the slow differentiation, or “dormancy,” of posterior structures given delayed hindlimb outgrowth^12^ and the steep decrease of the segmentation clock to form posterior somites.^92^ However, the early migration of the cranial neural crest^97^ and the early specification of the forelimb field^11,12^ also pointed to acceleration of anterior structures. Our transcriptomic data support accelerated progression of anterior features as a key factor driving anterior bias given that not only early specification of tissues but also early patterning and differentiation take place. The net effect is uncoupling of transcriptional and morphological progression, providing an interesting contrast to observations in eutherians. For instance, birth of post-mitotic neurons in the spinal cord can precede neural tube closure. Similarly, medial-lateral patterning of the limb field does not require limb outgrowth.

Species-specific differences in the time of initiation of developmental program have been attributed to *cis*-regulatory elements. For instance, the early expression of *SOX9* in the opossum is driven by a marsupial-specific sequence in a *SOX9* enhancer.^98^ Our finding that heterochrony is observed in ectoderm, mesoderm, and endoderm suggests that broader mechanisms may also be at play. However, different mechanisms could be involved in different tissues. Differences in the pace of development between species have been linked to fundamental cellular functions, including the rate of protein degradation and metabolism.^99–103^ However, it is unknown if these paradigms also apply to different tissues within the same organism. Our analyses indicate that the regulation of translation rates could be one of the processes controlling the pace of development in tissues within the same organism. The increase in protein synthesis rates in forelimbs and neural crest at the time of tissue prioritization suggests its implication in developmental heterochrony. When technical limitations allow, functional experiments in the opossum will be critical to further investigate the role of translation in heterochrony. A general mechanism may regulate translation rates within the embryo to achieve a compartmentalized response along the A-P axis. The mTOR pathway is a candidate sensor to promote these tissue-specific responses. Differences in growth factors, nutrient availability, and oxygen could be some of the elements that modulate mTOR activity. Lower translation rates, driven by reduced mTOR activity, are also associated with diapause, another physiological dormant state in mammals.^104,105^ In zebrafish, cell-type-specific response to temperature changes leads to loss of synchronicity, and this is mediated by proteostasis control.^106^ Hence, developmental progression and dormancy could be orchestrated by similar mechanisms across different contexts.

Our work highlights how cross-species comparative transcriptomics can provide insight not only into the conservation of cell lineages but also into temporal shifts. Our findings show that marsupials deploy conserved developmental programs but through a different temporal code. The datasets reported here provide a key resource for broader evolutionary analyses to delineate mammalian development.

### Limitations of the study

The cells included in the transcriptomic analysis constitute a fraction of those in the opossum embryo. We assume that these cells are a good representation of each time point, but we cannot rule out the possibility that certain cell populations are over- or underrepresented.

Cell-type annotation into discrete categories may be subject to inaccuracies when applied to cells undergoing continuous differentiation. These challenges are exacerbated in cross-species comparisons, and we cannot guarantee a one-to-one cell state equivalence. Additionally, some genes are missing or not annotated in the opossum genome, which means that some key markers’ expression was not used to annotate clusters and compare their expression patterns with other species. However, the results inferred by the single-cell analysis were supported by *in situ* stainings, suggesting that the high conservation of developmental programs across mammals minimizes any potential inaccuracies in cell-type annotation.

Finally, this study is limited to one species of marsupials, *Monodelphis domestica*. Work in other marsupials will be needed before we can extrapolate our findings to other species.

## Resource Availability

### Lead contact

Requests for further information and resources should be directed to and will be fulfilled by the lead contact, James Turner (james.turner@crick.ac.uk).

### Materials availability

This study did not generate unique reagents.

## Star★Methods

### Key Resources Table

**Table:** 

REAGENT or RESOURCE	SOURCE	IDENTIFIER
Antibodies		
Rabbit polyclonal anti phospho-S6 ribosomal protein (p-RPS6 Ser^240^/^244^)	Cell Signaling Technology	Cat#2215S; RRID:AB_331682
Rabbit polyclonal anti POU5F3	Niwaetal.^107^	N/A
Chemicals, peptides, and recombinant proteins		
Papain	Sigma-Aldrich	Cat#10108014001
Rock inhibitor	Stemcell Technologies	Cat#Y-27632
Critical commercial assays		
Click-iT® Plus OPP Alexa Fluor® 488 protein synthesis assay kit	Life Technologies	Cat#C10456
Chromium Next GEM Single Cell 3’ Kit v3.1	10x Genomics	Cat#PN-1000121
Single Index Kit T Set A	10x Genomics	Cat#PN-1000213
HCR RNA FISH	Molecular Instruments	https://www.molecularinstruments.com/
Deposited data		
Opossum sc-RNAseq data	This paper	GEO: GSE264385
Mouse sc-RNAseq data	Imaz-Rosshandler et al.^17^	https://marionilab.github.io/ExtendedMouseAtlas/
Rabbit sc-RNAseq data	Ton et al.^20^	https://marionilab.github.io/RabbitGastrulation2022/
Macaque sc-RNAseq data	Zhai et al.^23^	GEO: GSE193007
Experimental models: Organisms/strains		
Monodelphis domestica	The Francis Crick Institute	NA
Software and algorithms		
kallisto	Bray et al.^108^	https://www.kallistobus.tools/
DropletUtils	Lun et al.^109^	https://bioconductor.org/packages/release/bioc/html/DropletUtils.html
Seurat	Hao et al.^110^	https://satijalab.org/seurat/
Slingshot	Street et al.^111^	https://bioconductor.org/packages/devel/bioc/vignettes/slingshot/inst/doc/vignette.html
biomaRt	Durinck et al.^112^	https://www.ensembl.org/info/data/biomart/biomart_r_package.html
clusterProfiler	Yu et al.^113^	https://bioconductor.org/packages/release/bioc/html/clusterProfiler.html
Fiji	Schindelin et al.^114^	https://imagej.net/
GraphPad Prism	https://www.graphpad.com/	https://www.graphpad.com/

### Experimental Model and Study Participant Details

#### Opossum maintenance

Opossums (*Monodelphis domestica*) were maintained following the United Kingdom Animal Scientific Procedures Act 1986, and according to ethical guidelines at the Francis Crick Institute. Protocols were approved by local ethical review and the UK Home Office (Project Licence PP1234304). Opossums were housed in individual double-decker cages (GR1800, Tecniplast), with constant access to water and food, on a 14/10 h light/dark cycle. During nonbreeding periods, males and females were maintained in different rooms at 24-28°C and 55-75% humidity. For breeding, male and female opossums were kept next to each other in their individual cages for two days, and then swapped into each other’s cages for two days. Subsequently, they were put together into the same cage and breeding was monitored by CCTV, which allowed accurate estimates of developmental stages.

### Method Details

#### Opossum embryo recovery and single-cell dissociation

Embryos were recovered from the uterus of female opossums in posphate buffered saline (PBS) and kept on ice during processing. They were transferred to plates with HBSS (Life Technologies, 14175053) and 5% fetal bovine serum (FBS) for removal of the shell coat and most of the extraembryonic tissues (except for E8.5 embryos where extraembryonic tissues were kept). Several embryos from the same litters were pooled by stage (10 embryos for E8.5, 6 embryos for E9.5, 5 embryos for E10.0 and 2 embryos for E10.5) (Table S1). Opossum embryos from the same litter are broadly synchronised and morphologically very similar. To gain spatial resolution, embryos at E10.0 and E10.5 were divided into two parts along the A-P axis. Pooled samples were incubated on FACSmax Cell Dissociation Solution (Amsbio, T200100) with 10% Papain (30 U/mg, Sigma-Aldrich, 10108014001) for 11-12 min at 37°C to dissociate the cells as previously described.^84^ Samples were then resuspended in HBSS with 5% FBS, 10 μM rock inhibitor (Stemcell Technologies, Y-27632), and 1x non-essential aminoacids (Life Technologies, 11140050). Cells were filtered through 35 μm cell strainers (Falcon, 352235) and 20 μm filters (Miltenyi Biotec, 130-101-812). Samples with a cell viability above 60% were used for sequencing.

#### 10X Genomics single-cell RNA-sequencing and quantification

Single-cell suspensions were loaded into the 10X Genomics Chromium Chips and followed the manufacturer’s instructions for Chromium Next GEM Single Cell 3’ Kit v3.1 (PN-1000121). Libraries were prepared using the Single Index Kit T Set A (PN-1000213) and sequenced on the Illumina HiSeq4000 system or the Illumina NovaSeq6000 system.

Gene expression matrices were computed from FastQ files using the kallisto (0.46.2) bustools (0.40.0) workflow.^108,115^ A custom reference genome was created using the Ensembl ASM229v1 genome which was supplemented with a pseudo-Y chromosome using known *M. domestica* cDNAs from Y-chromosome genes.^28,116^ Similarly, Ensembl gene models from release-103 were supplemented with predicted gene models for the Y chromosome. Sequencing replicates were merged before quantification. For quantification by kb count, the ‘10xv3’ technology was specified and data output in Cell Ranger format using the ‘cellranger’ option.

Cells were identified from droplets by DropletUtils (1.12.1)^109^ using the equivalent threshold applied by Cell Ranger. Datasets were written in Cell Ranger format using the write10xCounts function.

#### Seurat analysis

Datasets were loaded into Seurat (4.0.3)^110^ objects for analysis in R (4.1.1). Initial quality control was applied to each sample independently using the number of genes identified, total UMI and percentage mitochondrial expression. The thresholds applied are listed in Table S1. A total of 23,669 cells were retained after filtering. For all the analyses performed in this work, we assume that our dataset maintains the cell type composition of the embryo.

Cell cycle was assessed in each cell using the CellCycleScoring function and homologues to the S and G2M genes included in the Seurat cc.genes list. The difference (S-G2M) was calculated and saved into the object’s metadata. Similarly, the AddModuleScore function was used to identify male cells based on the expression of two Y-linked genes broadly expressed at these time points: *RPL10Y* and *HMGB3Y*.

Datasets were normalised and scaled using NormalizeData and ScaleData for visualisation and calculation of module and cell cycle scores; and SCTransform, for datasets that would be integrated. The 3,000 most-variable genes selected by FindVariableFeatures using the ‘vst’ method were used by SCTransform. Both the cell cycle score and Y-gene score were regressed from the data using the ‘vars.to.regress’ parameter of SCTransform.

Anterior and posterior datasets were integrated within time points using the Seurat IntegrateData workflow and the SCTransformnormalised data.

PCA was applied to each of the samples and integrated datasets and the first 70 principle components retained. Dimensionality was estimated for each dataset and is detailed in Table S1. UMAP and tSNE reductions were subsequently calculated using RunUMAP and RunTSNE, respectively. Clusters were identified using the FindNeighbors and FindClusters functions at a range of resolutions.

Datasets were subsequently subset to select embryonic cells, removing extraembyonic cells from the data (except in the E8.5 dataset). Once subset, the variable features were identified, the data rescaled, PCA recalculated and dimensionality estimated. UMAP and tSNE reductions and clusters could then be identified in each cell subset.

Selected cluster representing specific cell types from different time points were subset and integrated to generate tissue-specific UMAPs.

#### Cluster annotation

We used gene module scores based on the combinatorial expression of conserved genes associated with specific cell states (Table S2) to identify the cell types present in our datasets. Module scores for cell types were calculated using AddModuleScore to help visualise cell types and select an appropriate cluster resolution for each dataset. A cluster resolution of 0.2 was used for E8.5 datset, a resolution of 0.8 was used for E9.5 and E10.0 dataset, and a resolution of 1.4 was used for E10.5.

The early mesoderm clusters from the E10.0 dataset expressed mesoderm-associated markers including *TBXT, SNAI1*/*2, HAND1*/*2* and *MESP1*^37,117–119^ but they did not have a clear signature of more mature subtypes. We took advantage of trajectory inference analyses using Slingshot^111^ (v2.14.0) to order these clusters and annotate them as nascent, early and advanced mesoderm (Figure S2D). Number of cells per time point and cluster are shown in Table S2.

#### Cross-species transcriptome-wide comparisons

Mouse,^17,19^ macaque^23^ and rabbit^20^ scRNA-seq datasets were downloaded and prepared for comparison to the opossum data. Each dataset was provided with cell type annotations which were kept for this analysis. Seurat objects were created for each dataset and only 1:1 orthologous genes, identified using the biomaRt package,^112^ were retained. For each species and time point, a set of variable genes were identified. For each time point comparison, the intersect between opossum and the comparison species’ variable genes were used to calculate Spearman’s rank correlation coefficient implemented by cor.test in R.

#### Cross-species comparisons through single-cell embedding

Neural crest, limb-associated and definitive endoderm cells were subset from mouse (E7.5, E8.0, E8.5, E9.0 and E9.5) and rabbit scRNA-seq datasets (GD8 and GD9). Neural crest cells labelled as “Migratory neural crest”, “Frontonasal neural crest” and “Brachial neural crest” in the mouse dataset, and labelled as “Migratory neural crest” and “Cranial neural crest” in the rabbit dataset were included in the analysis. Cells labelled as “Lateral plate mesoderm” and “Limb mesoderm” in both mouse and rabbit datasets were included in the analysis. Mouse definitive endoderm cells labelled as “Node”, “Gut tube”, “Pharyngeal endoderm”, “Foregut”, “Midgut” and “Hindgut” were included in the analysis.

A reference UMAP was built with each subset of cells for mouse and rabbit. Opossum neural crest (“pre-neural / neural crest”, “neural crest I” and “neural crest II”), limb-associated cells (“lateral plate mesoderm”, “mesenchyme forelimb” and “mesenchyme hindlimb”) and definitive endoderm cells from E10.0 and E10.5 were subset and embedded into the relevant mouse and rabbit reference UMAPs using the Seurat FindTransferAnchors, MapQuery workflow. The first 20 dimensions and 20 nearest neighbours (k.anchor) were used to identify transfer anchors. The TransferData function was subsequently used to predict cell type and time point of opossum cells using the mouse or rabbit labels. The number of opossum cells per predicted identity is shown in Table S4.

#### A-P priority score

A-P priority score was calculated based on the difference between the number of differentially expressed genes (DEG) between the anterior dataset of time point t_1_ vs timepoint t_0_ and the number of DEG between the posterior dataset of time point t_1_ vs time point t_0_ and divided by the number of DEG between whole embryos of time points t_1_ vs t_0_. The datasets used were: opossum E9.5, opossum E10.5 (anterior, posterior and whole embryos), mouse E7.5, mouse E9.0 (anterior, posterior and whole embryos), rabbit E7 and rabbit E9 (anterior, posterior and whole embryos). All datasets were downsampled to 2,000 cells using the subset function and different seed parameters to randomise the sampling up to ten times.

(Equation 1) $$\text{A}-\text{P}\;\text{priority}\;\text{score}\;=\frac{\text{DEG}\left[{\text{t}}_{1}^{\text{anterior}\;}\text{vs}\;{\text{t}}_{0}\right]-\text{DEG}\left[{\text{t}}_{\text{1}}^{\text{posterior}\;}{\text{vs}\;\text{t}}_{\text{0}}\right]}{\text{DEG}\left[{\text{t}}_{\text{1}}{\;\text{vs}\;\text{t}}_{\text{0}}\right]}$$

The number of DEG was calculated using the Seurat FindMarkers function (logfc.threshold = 0, min.pct = 0.005) from Seurat.^110^ An adjusted p-value <0.05 was considered to identify DEG. The number of DEG per comparison is shown in Table S3.

For tissue-specific A-P priority score calculations, the following annotated cell clusters were subset. For endoderm, opossum cluster annotated as “definitive endoderm” (downsampled to 30 cells per dataset), mouse clusters annotated as “Node”, “Gut tube”, “Pharyngeal endoderm”, “Foregut”, “Midgut” and “Hindgut” (downsampled to 150 cells per dataset), and rabbit clusters annotated as “Definitive Endoderm”, “Gut tube”, “Gut endoderm”, and “Pharyngeal endoderm” (downsampled to 180 cells per dataset) were used. For non-neural ectoderm, opossum cluster annotated as “non-neural ectoderm” (downsampled to 170 cells per dataset), mouse clusters annotated as “Non-neural ectoderm”, “Surface ectoderm”, “Limb ectoderm”, “Ectoderm” and “Placodal ectoderm” (downsampled to 350 cells per dataset), and rabbit clusters annotated as “Non-neural ectoderm 2”, “Non-neural ectoderm 4”, and “Placodal ectoderm” (downsampled to 50 cells per dataset) were used. For neural ectoderm, opossum cluster annotates as “neural tube” (downsampled to 270 cells per dataset), mouse clusters annotated as “Epiblast”, “Neural tube”, “Early dorsal fore-brain progenitors”, “Late dorsal forebrain progenitors”, “Ventral forebrain progenitors”, “Midbrain progenitors”, “Midbrain/ Hindbrain boundary”, “Hindbrain neural progenitors”, “Dorsal hindbrain progenitors”, “Ventral hindbrain progenitors”, “Hindbrain floor plate”, “Optic vesicle”, “Otic neural progenitors”, “Spinal cord progenitors”, “Dorsal spinal cord progenitors” (downsampled to 300 cells per dataset), and rabbit clusters annotated as “Epiblast”, “Forebrain”, “Midbrain”, “Hindbrain”, “Spinal cord”, “Roof plate”, and “Floor plate” (downsampled to 200 cells per dataset) were used. The same time points as before were used (E7.5 and E10.5 for opossum, E7.5 and E9 for mouse, and E7 and E9 for rabbit). In the case of rabbit endoderm, the anterior section was compared to the middle section instead of the posterior section because there were only 8 cells annotated as endoderm in the posterior section.

#### Opossum gene expression comparisons

Gene expression comparisons between specific clusters of the opossum dataset were performed using Seurat FindMarkers function (logfc.threshold = 0.01). Neural crest from E10.5 anterior and posterior datasets were downsampled to 350 cells to equalise the number of cells. Genes enriched in anterior (positive log_2_ fold change) and posterior (negative log_2_ fold change) are shown in Table S5.

Similarly, forelimb and hindlimb clusters from E10.5 dataset were downsampled to 400 cells. Genes enriched in forelimb (positive log_2_ fold change) and hindlimb (negative log_2_ fold change) are shown in Table S5.

Spinal cord cells from E10.5 anterior and posterior datasets were downsampled to 500 cells and the expression of key markers was compared by Student’s t-test in both populations.

Subclusters in E10.5 definitive endoderm cells were found using the Seurat FindSubCluster function. Genes enriched in each sub-cluster are shown in Table S5.

#### Gene set enrichment analysis

Enrichment of biological processes in forelimb cells (Table S5) was done using the gseGO() function, as part of the R package clusterProfiler^113^ (v4.2.2). Log_2_ fold change between forelimb and hindlimb was used as ranking metric, specifying the Biological Process (BP) ontology database.

#### Pseudotime trajectory analysis

Lateral plate mesoderm, mesenchyme forelimb, and mesenchyme hindlimb clusters from E10 and E10.5 datasets were subset and re-integrated. After re-clustering at resolution 0.2, we verified that cells were still grouped according to their cell type identity. We used Slingshot^111^ (v2.14.0) to infer pseudotime lineage trajectories using lateral plate mesoderm as the starting cluster.

Pseudotime trajectory analysis could be applied to limbs but was unreliable for comparing anterior and posterior neural crest, spinal cord or endoderm. This is because A-P transcriptional differences of these three tissues are less defined at this stage, and the integration of A and P datasets to mitigate batch effects masked these differences.

#### Immunofluorescence

Opossum E10.5 embryos were dissected in Dulbecco’s phosphate buffer saline (DPBS) (Thermo Fisher Scientific, 14190094) and fixed with 4% paraformaldehyde (PFA) for 2 hours at room temperature. Embryos were incubated in 0.5% Triton X-100 (Sigma, 93443) in PBS (PBST) for 1 hour at room temperature for permeabilization and in 2% bovine serum albumin (BSA) at 4°C overnight for blocking. Embryos were incubated in the primary antibody phospho-S6 ribosomal protein (p-RPS6 Ser^240/244^; 1:100 in 2% BSA, Cell Signalling Technology, 2215S) for 3 days at 4°C. After multiple washes in PBST, embryos were incubated with secondary antibodies coupled to specific fluorophores (1:500 in 2% BSA, Invitrogen) overnight at 4°C. Nuclei were stained with DAPI (1:1000 in PBST, Sigma, D9564) incubation, for 1 hour at room temperature. Embryos were mounted on SuperFrost™Plus microscope slides using Vectashield mounting medium (Vector laboratories, H-1000-10).

#### HCR RNA-FISH

Fluorescent *in situ* hybridization chain reaction (HCR RNA-FISH) was performed using commercial reagents (Molecular Instruments) following the v3 protocol for whole-mount mouse embryos with some modifications.^120^ Briefly, embryos were fixed in 4% paraformaldehyde (PFA) for 2 hours at room temperature, dehydrated in a methanol series and stored at -20 °C until used. After rehydration, embryos were treated with proteinase-K (10 μg/mL) for 10 min at room temperature and post-fixed with 4% PFA for 20 min at room temperature. Embryos were then incubated with probe hybridization buffer for 30 min at 37 °C. Probes, designed by Molecular Instruments, were prepared at 4 pmol/mL in hybridization buffer. Embryos were incubated in the probe solution at 37 °C overnight. Excess probes were removed by washing embryos in probe wash buffer at 37 °C followed by washes in SSCT (5X sodium chloride sodium citrate, 0.1% Tween-20) at room temperature. Hairpins were prepared by snap-cooling at 95 °C for 90 s and cooled to room temperature protected from light and diluted in amplification buffer (60 pmol/mL). After 5 min incubation in amplification buffer, embryos were incubated in hairpin solution overnight at room temperature. Excess hairpins were removed by washing in SSCT. Nuclei were stained by DAPI and samples were mounted on slides using vectashield (Vector laboratories, H-1000-10). At least two opossum embryos were used per experiment.

HCR immunofluorescence combined with RNA-FISH was performed following the v3 HCR 2° IHC + HCR RNA-FISH protocol for samples in solution (Molecular Instruments). The antibodies used were POU5F3 (gift from H. Niwa,^107^ 1:200) and p-RPS6 Ser^240/244^ (1:100, Cell Signalling Technology, 2215S).

#### Protein synthesis labelling

O-propargyl-puromycin (OPP) was used to detect nascent protein synthesis in opossum embryos. Click-iT® Plus OPP Alexa Fluor® 488 protein synthesis assay kit (Life Technologies, C10456). Embryos were incubated in 10 μM OPP in DMEM-GlutaMax (ThermoFisher, 31966021) supplemented with 20% fetal bovine serum (Gibco, 01098) for 1 hour in a standard CO_2_ incubator at 32.6°C. After fixation in 4% PFA for 2 hours at room temperature, Click-iT reaction was performed according to manufacturer’s instructions.

When combined with HCR RNA-FISH, samples were dehydrated in methanol series after fixation in 4% PFA and the HCR RNA-FISH protocol was subsequently followed as usual. Click-iT reaction for OPP was performed after the HCR RNA-FISH protocol was completed.

#### Cryosectioning

After whole-mount HCR RNA-FISH, selected opossum embryos were embedded in OCT and snap frozen. Transversal sections (15-20 μm thickness) were taken along the A-P axis and put onto Superfrost Plus slides (Thermo Scientific).

Selected opossum embryos incubated in 20 μM OPP and used for HCR RNA-FISH were embedded in OCT and snap frozen. Lon-gitudinal sections (10 μm thickness) were taken along the D-V axis. Click-iT reaction to detect OPP was performed after cryosectioning following manufacturer’s instructions.

#### Imaging

Whole-mount and sectioned embryos were imaged using a 40x immersion lens and a 20x lens on an Olympus SoRa Spinning disc. Z-stacks were collected at 3-4 μm intervals, and tile scanning was used. Images were processed using Fiji.^114^ Optical sections, maximum intensity or Sum slices Z-projections are shown.

### Quantification and Statistical Analysis

#### Image analysis

HCR RNA-FISH, OPP labelling and p-RPS6 immunofluorescence images were analysed using Fiji.

*LMX1B, ZIC2* and *TFAP2C* levels were quantified in transverse forelimb sections of E10.5 opossum embryos. The forelimb field was split into medial and lateral domains, and the expression intensity levels of each marker were quantified in both domains. A total of 27 forelimb sections corresponding to 2 embryos were quantified. A ratio was then calculated by dividing the intensity levels of the lateral domain by the levels of the medial domain.

*NKX6-1* and *MSX1* intensity levels were quantified along the A-P axis of the spinal cord in E10.5 embryos. “Sum slice” Z-projections of 6-9 optical sections covering dorsal or ventral regions were generated. 21 measurements were taken along the A-P axis for *NKX6-1* (ventral region) and *MSX1* (dorsal region). Both sides of the spinal cord were measured separately, and two embryo were used. The measurements were normalised by the average expression of each marker in each embryo. Normalised intensity levels were plotted using GraphPad Prism 10.

OPP and p-RPS6 intensity levels were measured in small regions along the A-P axis in the same optical section. Each measurement corresponded to the average intensity levels of a defined area. Neural crest and spinal cord regions were delimited by the presence or absence of *SOX9* respectively. Forelimbs were identified by the expression of *TBX5*. The measurements were normalised by the average expression of OPP and p-RPS6 in each embryo and separating left and right sides. 32 measurements were taken among two different embryos for OPP staining and 46 measurements were taken among three different embryos for p-RPS6. Normalised intensity levels were plotted using GraphPad Prism 10. One-way ANOVA with Tukey’s multiple comparison tests were performed to compare intensity levels across the three regions. To account for potential differences in cell density, we repeated the analysis to normalise OPP levels by cell density (by manually counting nuclei identified with DAPI staining). Normalised intensity levels were plotted using GraphPad Prism 10 and p-values were calculated using RM one-way ANOVA and Fisher’s Least Significant Difference (LSD) test.

OPP levels in forelimb (*TBX5*-positive) and hindlimb (*PITX1*-positive) domains were quantified in longitudinal sections in which both domains were present. Apart from the heart, all *TBX5* labelled areas were quantified independently of their position along the A-P axis. The “Sum slice” Z-projection was generated for each section prior to quantification, and several measurements were taken for each domain. Measurements corresponded to the average intensity levels of a defined area. To assess relative changes, each measurement was normalised to the average OPP intensity across the whole domain (e.g., forelimb + hindlimb). This allowed us to determine whether OPP intensity in each region was enriched or depleted relative to the domain average. 8 longitudinal sections from 2 embryos were used. To account for potential differences in cell density, we repeated the analysis to normalise OPP levels by cell density (by manually counting nuclei identified with DAPI staining). Normalised intensity levels were plotted using GraphPad Prism 10 and p-values were calculated using a Student’s t-test.

#### Statistical analysis

Different statistical tests were performed to analyse specific data. Spearman’s rank correlation coefficients were used in Figures 1C, S1D, S1E, and S1F. One-way ANOVA with Tukey’s multiple comparison test was used in Figures 1D, 1E, 7A, and 7F. RM one-way ANOVA with Fisher’s LSD test was used in Figure S7B. Student’s t test was used in Figures 3F, 5B, 7D, and S5D. Mann-Whitney t-test was used in Figure S7B and S7H.

## Supplementary Material

Supplemental information can be found online at https://doi.org/10.1016/j.devcel.2025.06.037.

Supplemental Information

Table S1

Table S2

Table S3

Table S4

Table S5

Table S6

## Data Availability

scRNA-seq data generated from this study have been deposited in the National Center for Biotechnology Information’s Gene Expression Omnibus database under accession code GEO: GSE264385. Loupe files to visualize and explore the datasets using the Loupe Browser (10× Genomics) are included. This study does not report original code. Any additional information required to reanalyze the data reported in this work paper is available from the lead contact upon request.

## Highlights

- Single-cell transcriptomic atlas of opossum gastrulation and early organogenesis
- Heterochrony is evident in neural crest, limbs, spinal cord, and endoderm
- Opossum embryos exhibit uncoupling of transcriptional and morphological timelines
- Translation control is a candidate mechanism behind heterochrony

## In brief

Menchero et al. generate a single-cell transcriptomic atlas in the opossum and show rapid progression of transcriptional programs in specific tissues relative to morphological landmarks. This shift in timing, or heterochrony, adapted to the extremely short gestation of marsupials, could be mediated by translational regulation.
