# Supplementary material for: Marsupial single-cell transcriptomics identifies temporal diversity in mammalian developmental programs
Source: Dev Cell. Author manuscript; Available in PMC 2026 Oct 22. (PMC7619616; doi:10.1016/j.devcel.2025.06.037)
Supplement: Supplementary file 1 [file EMS219627-supplement-Supplemental_Information.pdf]

Developmental Cell, Volume *60*

Supplemental information

Marsupial single-cell transcriptomics identifies temporal diversity in mammalian developmental programs

Sergio Menchero, Christopher Barrington, Clare Critcher, Wazeer Varsally, Gregorio Alanis-Lobato, Kathy K. Niakan, and James M.A. Turner

Figure S1

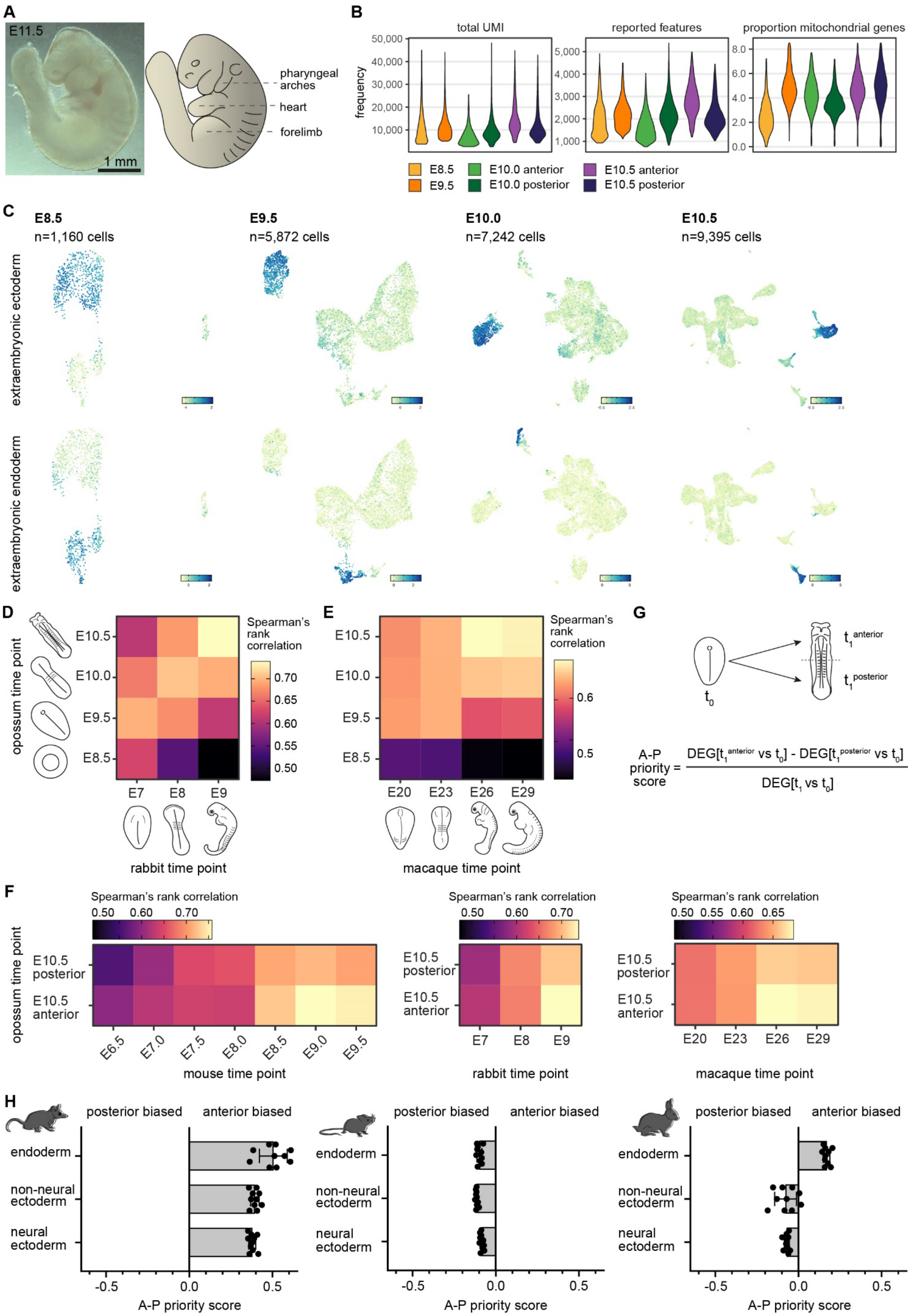
A
E11.5
1 mm
pharyngeal arches
heart
forelimb
B
total UMI
reported features
proportion mitochondrial genes
frequency
E8.5
E9.5
E10.0 anterior
E10.0 posterior
E10.5 anterior
E10.5 posterior
C
E8.5
n=1,160 cells
E9.5
n=5,872 cells
E10.0
n=7,242 cells
E10.5
n=9,395 cells
extraembryonic ectoderm
extraembryonic endoderm
D
opossum time point
Spearman's rank correlation
rabbit time point
E
Spearman's rank correlation
macaque time point
G
t_0
$t_1^{anterior}$
$t_1^{posterior}$
A-P priority score = $\frac{DEG[t_1^{anterior} \text{ vs } t_0] - DEG[t_1^{posterior} \text{ vs } t_0]}{DEG[t_1 \text{ vs } t_0]}$
F
Spearman's rank correlation
opossum time point
E10.5 posterior
E10.5 anterior
mouse time point
rabbit time point
macaque time point
H
posterior biased
anterior biased
endoderm
non-neural ectoderm
neural ectoderm
A-P priority score

Figure S1: Characterisation of the opossum single-cell RNA-sequencing dataset. Related to Figure 1.

- **A.** Stereo microscope image (left) and schematic illustration (right) of an E11.5 opossum embryo. Scale bar, 1 mm.
- **B.** Distribution of number of UMI, number of features and percentage of mitochondrial genes in each single-cell dataset after QC filtering.
- **C.** Feature plots of opossum UMAP by time point showing expression of module scores associated with extraembryonic ectoderm (D) and extraembryonic endoderm (E). Module scores are based on the combinatorial expression of markers reported in Table S2.
- **D.** Heatmap of Spearman's rank correlation coefficients of gene expression per opossum (vertical) and rabbit (horizontal) time point.
- **E.** Heatmap of Spearman's rank correlation coefficients of gene expression per opossum (vertical) and macaque (horizontal) time point.
- **F.** Heatmap of Spearman's rank correlation coefficients of gene expression per opossum E10.5 anterior and posterior datasets versus mouse (left panel), rabbit (middle panel) and macaque (right panel) datasets by time point. The intersect of variable features across timepoints between both species is used to calculate the correlation in all comparisons (A, B and C).
- **G.** A-P priority score diagram. A-P priority score is calculated based on the number of differentially expressed genes (DEG) between the anterior dataset of time point t_1 vs timepoint t_0 minus the number of DEG between the posterior dataset of time point t_1 vs time point t_0 and divided by the number of DEG between whole embryos of time points t_1 vs t_0.
- **H.** A-P priority score in endoderm, non-neural ectoderm and neural ectoderm for opossum (t0 = E9.5; t1 = E10.5), mouse (t0 = E7.5; t1 = E9.0) and rabbit (t0 = E7; t1 = E9) embryos. The score was calculated 10 times per tissue and species after random downsampling. The lack of anterior bias in mouse and rabbit suggests that the posterior transcriptomic landscape has progressed considerably from late gastrulation to early organogenesis in these species, while in the opossum, it has not. Data are represented as mean ± SD.

Figure S2

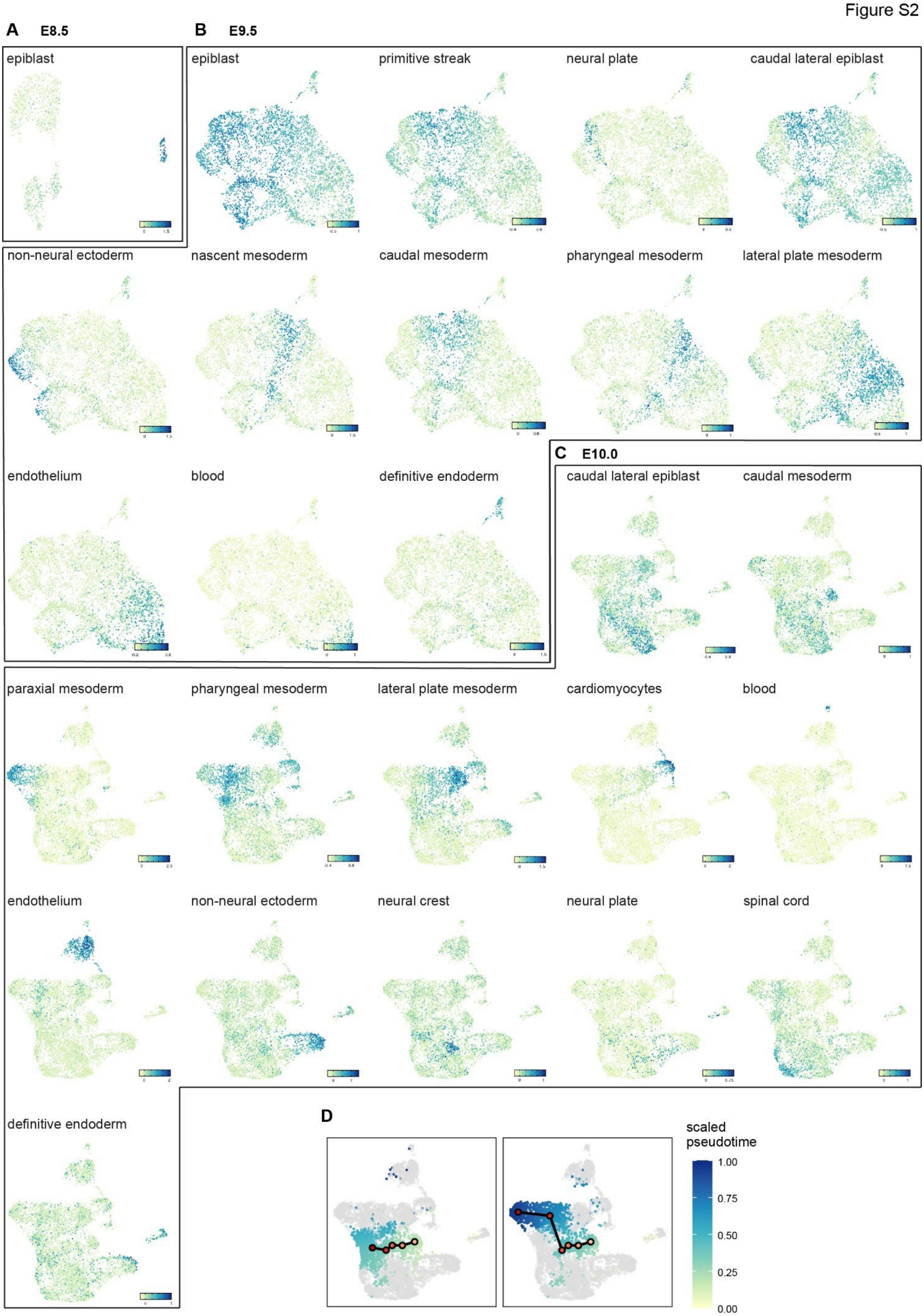
A E8.5
epiblast
B E9.5
epiblast
primitive streak
neural plate
caudal lateral epiblast
non-neural ectoderm
nascent mesoderm
caudal mesoderm
pharyngeal mesoderm
lateral plate mesoderm
endothelium
blood
definitive endoderm
C E10.0
caudal lateral epiblast
caudal mesoderm
paraxial mesoderm
pharyngeal mesoderm
lateral plate mesoderm
cardiomyocytes
blood
endothelium
non-neural ectoderm
neural crest
neural plate
spinal cord
definitive endoderm
D
scaled pseudotime
1.00
0.75
0.50
0.25
0.00

Figure S2: Cluster annotation based on module scores in E8.5, E9.5 and E10.0. Related to Figure 2.

- **A.** Feature plot of opossum E8.5 UMAP showing expression of module score associated with epiblast cells.
- **B.** Feature plots of opossum E9.5 UMAP showing expression of module scores associated with selected cell types. Module scores are based on the combinatorial expression of markers reported in Table S2.
- **C.** Feature plot of opossum E10.0 UMAP showing expression of module score associated with selected cell types. Module scores are based on the combinatorial expression of markers reported in Table S2.
- **D.** Opossum E10.0 UMAP showing pseudotime of early mesoderm clusters. Cells from selected clusters are coloured in a gradient from yellow to dark blue to illustrate pseudotime. The average pseudotime of each cluster is also represented with a circle coloured from yellow (early) to red (late).

Figure S3

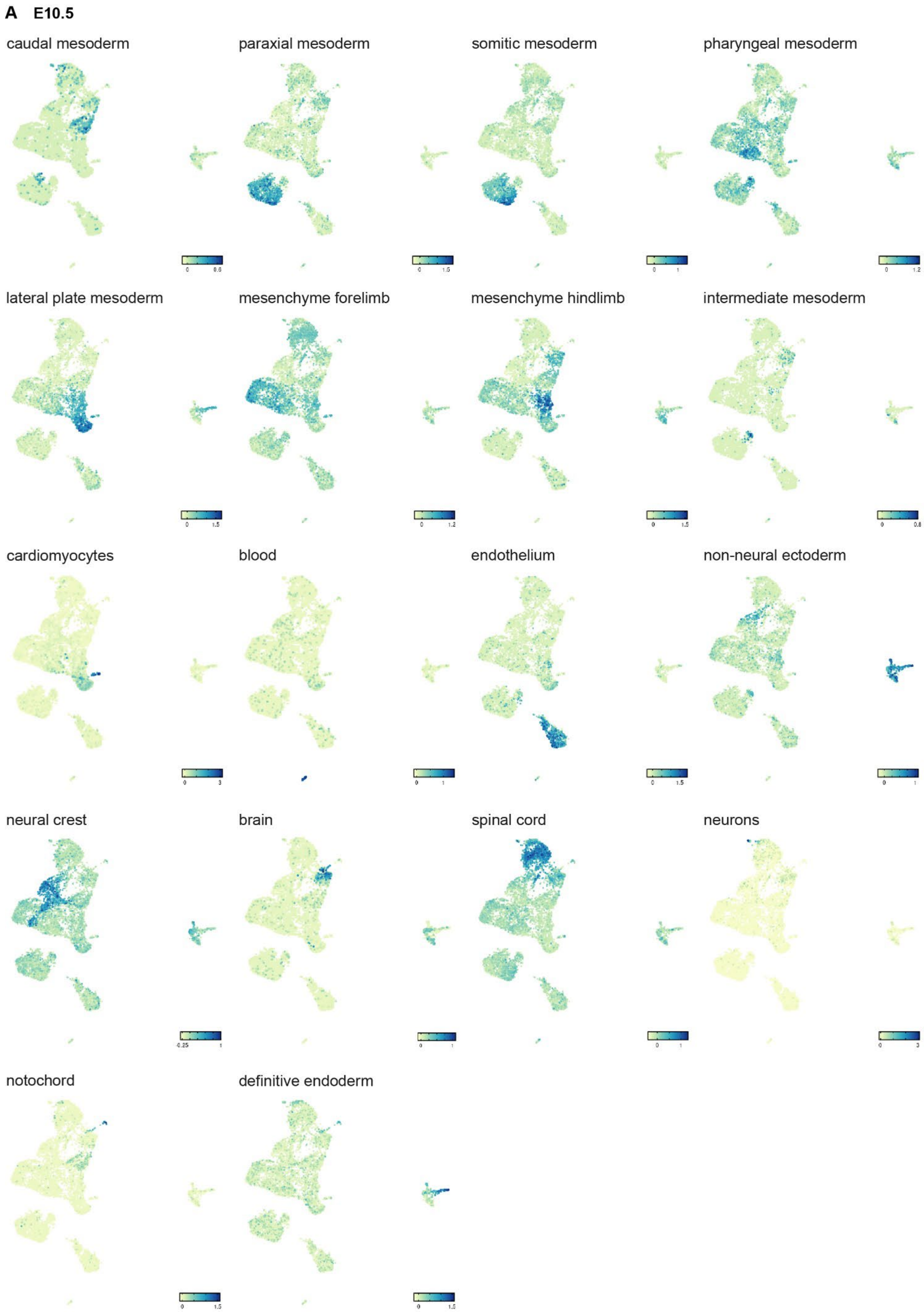
A E10.5
caudal mesoderm
paraxial mesoderm
somitic mesoderm
pharyngeal mesoderm
lateral plate mesoderm
mesenchyme forelimb
mesenchyme hindlimb
intermediate mesoderm
cardiomyocytes
blood
endothelium
non-neural ectoderm
neural crest
brain
spinal cord
neurons
notochord
definitive endoderm

Figure S3: Cluster annotation based on module scores in opossum E10.5. Related to Figure 2.

A. Feature plot of opossum E10.5 UMAP showing expression of module score associated with selected cell types. Module scores are based on the combinatorial expression of markers reported in Table S2.

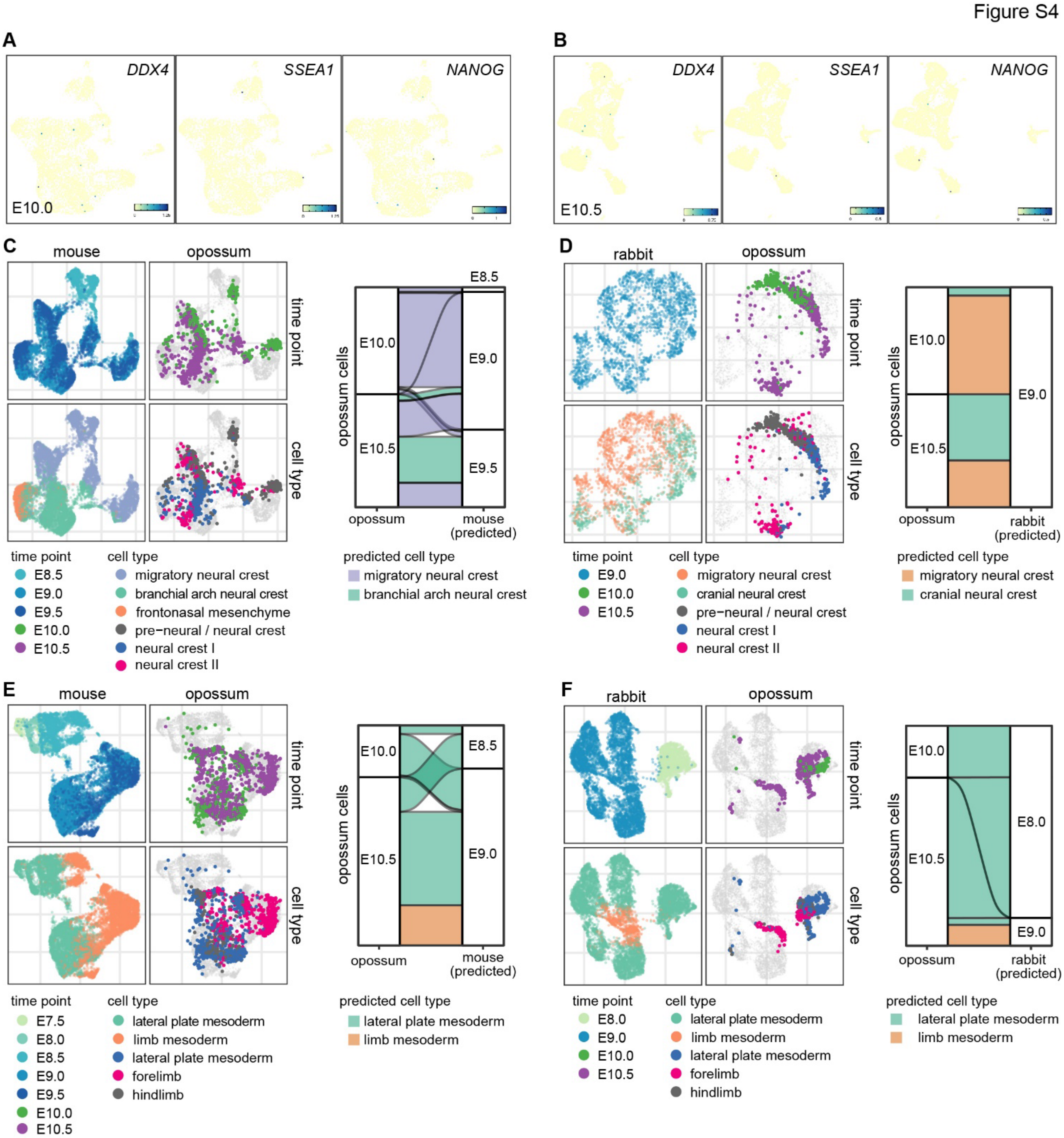

Figure S4. Cross-species sigle-cell embedding analysis. Related to Figure 2.

- **A.** Feature plots of UMAPs at E10 showing expression of PGC-associated markers: *DDX4*, *SSEA1* (also known as *FUT4*) and *NANOG.*
- **B.** Feature plots of UMAPs at E10.5 showing expression of the same genes.
- **C-D**. Mouse (C) and rabbit (D) reference neural crest UMAP and embedded opossum cells coloured by time point and cell type. Alluvial plots showing the predicted time point and cell type of opossum cells are shown on the right.
- **E-F**. Mouse (E) and rabbit (F) reference limb UMAP and embedded opossum cells coloured by time point and cell type. Alluvial plots showing the predicted time point and cell type of opossum cells are on the right.

Figure S5

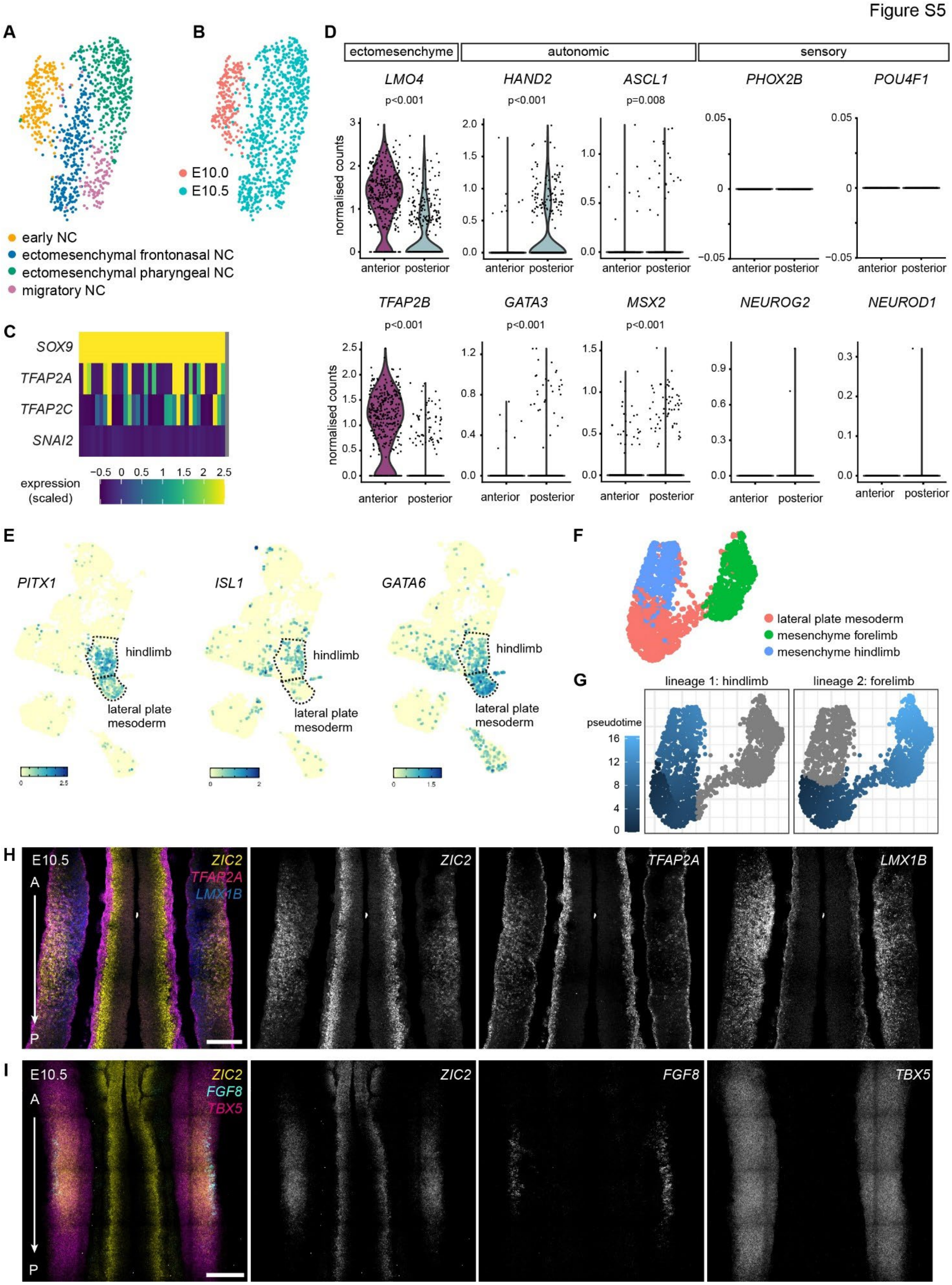

A
early NC
ectomesenchymal frontonasal NC
ectomesenchymal pharyngeal NC
migratory NC
B
E10.0
E10.5
C
SOX9
TFAP2A
TFAP2C
SNAI2
expression (scaled)
D
ectomesenchyme
autonomic
sensory
LMO4
HAND2
ASCL1
PHOX2B
POU4F1
p<0.001
p=0.008
normalised counts
anterior
posterior
TFAP2B
GATA3
MSX2
NEUROG2
NEUROD1
E
PITX1
ISL1
GATA6
hindlimb
lateral plate mesoderm
F
lateral plate mesoderm
mesenchyme forelimb
mesenchyme hindlimb
G
lineage 1: hindlimb
lineage 2: forelimb
pseudotime
H
E10.5
ZIC2
TFAP2A
LMX1B
A
P
I
E10.5
ZIC2
FGF8
TBX5
A
P

Figure S5: Neural crest and forelimb in the opossum embryo. Related to Figure 3 and Figure 4.

- **A.** UMAP of anterior neural crest cells from E10.0 and E10.5 embryos. Cluster annotation based on the expression of key marker genes as shown in Figure 3A.
- **B.** Umap of anterior neural crest cells coloured by time point.
- **C.** Heatmap of expression of neural crest markers in *SOX9* positive cells at E9.5.
- **D.** Violin plots for the expression of neural crest markers associated with ectomesenchyme, autonomic and sensory neural crest fate. P-values after Student's t test are shown when statistically significant ($p<0.05$).
- **E.** Feature plot of UMAP of selected E10.5 data showing expression of *PITX1*, *ISL1* (both associated with presumptive hindlimb field) and *GATA6* (lateral plate mesoderm).
- **F.** Integrated UMAP of opossum scRNA-seq data from E10 and E10.5 corresponding to lateral plate mesoderm, mesenchyme forelimb and mesenchyme hindlimb cell clusters.
- **G.** Pseudotime lineage trajectories identified with Slingshot in the integrated object.
- **H.** Expression of *ZIC2*, *TFAP2A* and *LMX1B* by HCR RNA-FISH in the forelimb domain of an opossum E10.5 embryo. *ZIC2* and *TFAP2A* expression occupies the most lateral domain of the forelimb field, while *LMX1B* is enriched in the medial domain. The image is an optical section. Scale bar, 200 μm. A = anterior; P = posterior.
- **I.** Expression of *TBX5*, *ZIC2* and *FGF8* by HCR RNA-FISH in the forelimb domain of an opossum E10.5 embryo. *FGF8* is expressed in the limb ectoderm. Image is a maximal projection. Scale bar, 200 μm. A = anterior, P = posterior.

Figure S6

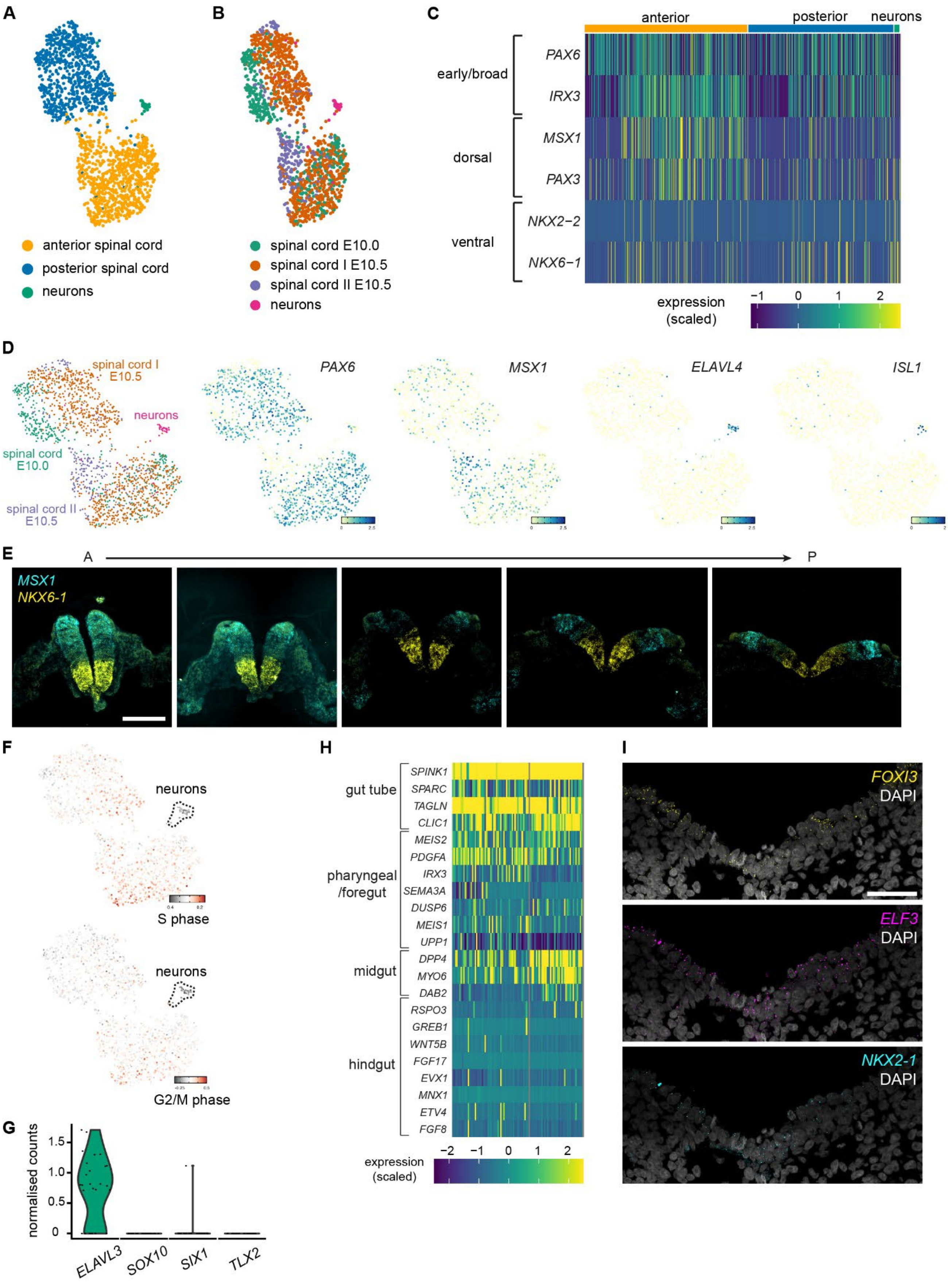

A
anterior spinal cord
posterior spinal cord
neurons
B
spinal cord E10.0
spinal cord I E10.5
spinal cord II E10.5
neurons
C
anterior
posterior
neurons
early/broad
PAX6
IRX3
dorsal
MSX1
PAX3
ventral
NKX2−2
NKX6−1
expression (scaled)
D
spinal cord I E10.5
neurons
spinal cord E10.0
spinal cord II E10.5
PAX6
MSX1
ELAVL4
ISL1
E
A
P
MSX1
NKX6-1
F
neurons
S phase
neurons
G2/M phase
G
normalised counts
1.5
1.0
0.5
0
ELAVL3
SOX10
SIX1
TLX2
H
gut tube
SPINK1
SPARC
TAGLN
CLIC1
pharyngeal /foregut
MEIS2
PDGFA
IRX3
SEMA3A
DUSP6
MEIS1
UPP1
midgut
DPP4
MYO6
DAB2
hindgut
RSPO3
GREB1
WNT5B
FGF17
EVX1
MNX1
ETV4
FGF8
−2
−1
0
1
2
expression (scaled)
I
FOXI3
DAPI
ELF3
DAPI
NKX2-1
DAPI

Figure S6: Spinal cord and endoderm in the opossum embryo. Related to Figure 5 and Figure 6.

- **A.** UMAP of spinal cord cells and neurons from E10.0 and E10.5 embryos. Cluster annotation based on the expression of *HOX* genes as shown in Fig. 5A.
- **B.** UMAP of spinal cord cells and neurons coloured by cluster of origin in E10.0 and E10.5 datasets.
- **C.** Heatmap of expression of selected genes associated with early/broad (*PAX6*, *IRX3*), dorsal (*MSX1*, *PAX3*) and ventral (*NKX2-2*, *NKX6-1*) populations in spinal cord clusters (yellow = anterior spinal cord; blue = posterior spinal cord; green = neurons).
- **D.** Feature plots of spinal cord and neurons UMAP showing expression of key genes associated with early spinal cord (*PAX6*), dorsal spinal cord (*MSX1*), pan neurons (*ELAVL4*) and motor neurons (*ISL1*).
- **E.** Transversal sections in five different regions along the A-P axis (from left to right) showing restricted expression of *MSX1* and *NKX6-1* by HCR RNA-FISH at E10.5. Scale bar, 50 µm.
- **F.** Feature plot of spinal cord and neurons UMAPs showing expression of module score associated with the S phase (top) and G2/M phase (bottom) of the cell cycle.
- **G.** Violin plots showing expression of pan-neuronal (*ELAVL3*) and neural crest-derived neuronal markers (*SOX10*, *SIX1*, *TLX2*) in opossum neurons at E10.5.
- **H.** Heatmap of expression of genes associated with gut tube, pharyngeal/foregut, midgut and hindgut domains in the definitive endoderm cells of E10.5 embryos.
- **I.** Expression of *FOXI3*, *ELF3* and *NKX2-1* by HCR RNA-FISH in the ventral foregut endoderm of an opossum E10.5 embryo. Image is a maximal projection of a cross section. Scale bar, 50 µm.

Figure S7

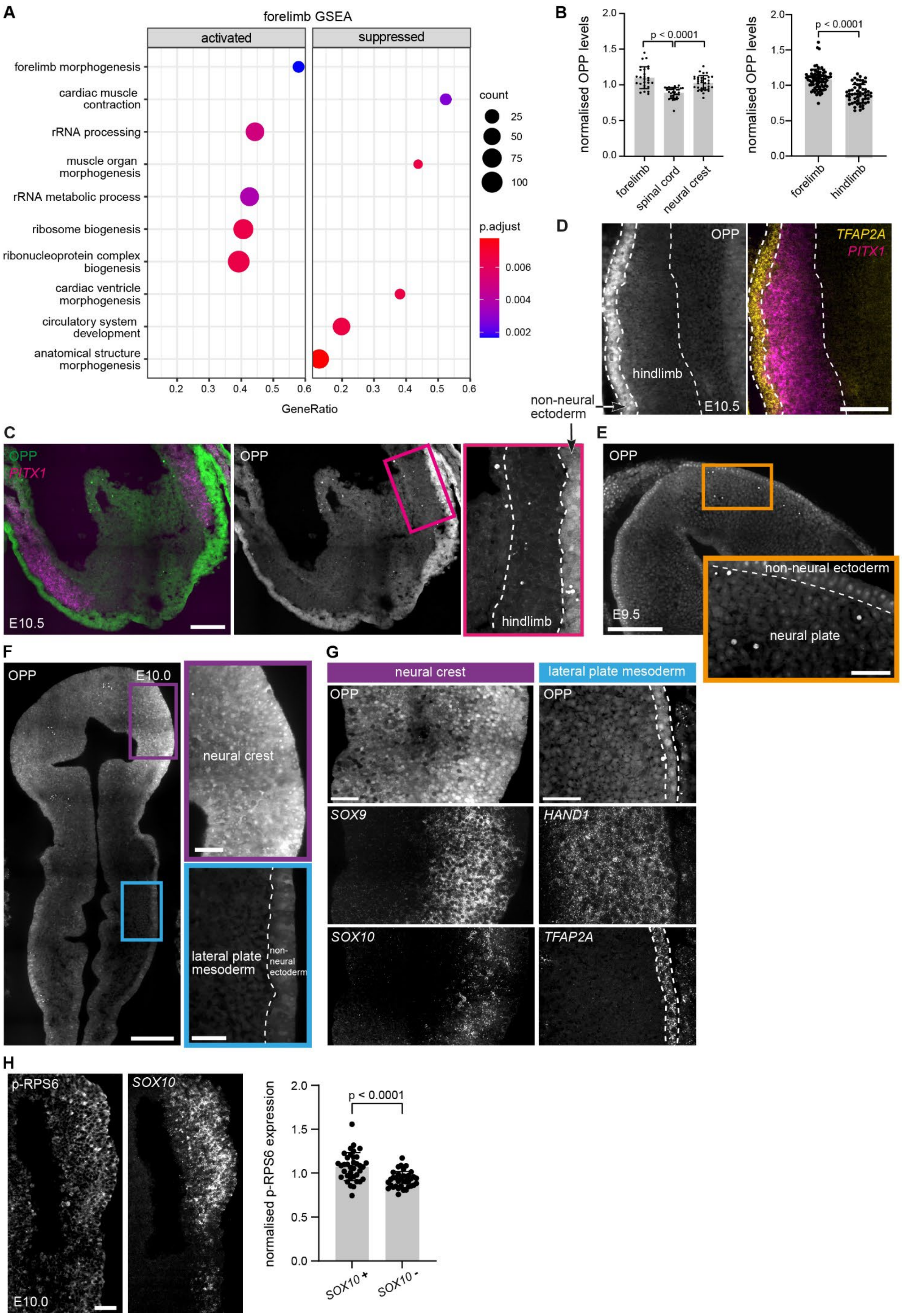
A
forelimb GSEA
activated
suppressed
forelimb morphogenesis
cardiac muscle contraction
rRNA processing
muscle organ morphogenesis
rRNA metabolic process
ribosome biogenesis
ribonucleoprotein complex biogenesis
cardiac ventricle morphogenesis
circulatory system development
anatomical structure morphogenesis
GeneRatio
count
25
50
75
100
p.adjust
0.006
0.004
0.002
B
normalised OPP levels
p < 0.0001
forelimb
spinal cord
neural crest
hindlimb
C
OPP
PITX1
E10.5
hindlimb
non-neural ectoderm
D
OPP
TFAP2A
PITX1
hindlimb
E10.5
E
OPP
E9.5
non-neural ectoderm
neural plate
F
OPP
E10.0
neural crest
lateral plate mesoderm
non-neural ectoderm
G
neural crest
lateral plate mesoderm
OPP
SOX9
HAND1
SOX10
TFAP2A
H
p-RPS6
SOX10
E10.0
normalised p-RPS6 expression
p < 0.0001
SOX10 +
SOX10 -

Figure S7: Protein synthesis enrichment in opossum forelimb and neural crest. Related to Figure 7.

- **A.** Gene set enrichment analysis in the transcriptome of the forelimb field in comparison with the hindlimb field. The top 5 activated and suppressed gene terms are shown.
- **B.** Comparison of normalised OPP levels by area. (left) Boxplot showing OPP levels measured along the A-P axis in forelimb (*TBX5* positive), neural crest (*SOX9* positive) and spinal cord regions. n=32 measurements (2 embryos). (right) Boxplot showing OPP levels measured in forelimb (*TBX5* positive) and hindlimb (*PITX1* positive) domains. Measurements were taken in different regions of 8 longitudinal sections from 2 embryos. P-value after RM one-way ANOVA and Fisher's LSD test (left plot) and Mann-Whitney t-test (right plot). Data are represented as mean ± SD.
- **C.** Longitudinal section in an opossum E10.5 embryo showing *PITX1* expression by HCR RNA-FISH and OPP labelling. Zoom-in area of OPP labelling in hindlimb is shown on the right panel. Scale bar, 100 µm
- **D.** Optical section in an opossum E10.5 embryo showing *TFAP2A* (non-neural ectoderm) and *PITX1* (hindlimb) expression by HCR RNA-FISH and OPP labelling. Scale bar, 100 µm
- **E.** OPP labelling in an opossum E9.5 embryo. The image is an optical section. Scale bar, 200 µm. Zoom-in area of OPP labelling in anterior neural plate is shown. Scale bar, 50 µm.
- **F.** OPP labelling in an opossum E10.0 embryo. The image is an optical section. Scale bar, 200 µm. Zoom-in areas of OPP labelling in the neural crest region and lateral plate mesoderm are shown on the right panel. Scale bar, 50 µm.
- **G.** Optical section in an opossum E10.0 embryo showing OPP labelling in neural crest cells (left), identified by *SOX9* and *SOX10* expression; and lateral plate mesoderm and non-neural ectoderm (right), identified by *HAND1* and *TFAP2A* respectively. Scale bar, 50 µm.
- **H.** Optical section in an opossum E10.0 embryo showing p-RPS6 and *SOX10* expression by HCR IF + RNA FISH. Scale bar, 50 µm. On the right panel, a boxplot showing p-RPS6 levels measured in neural crest (*SOX10* positive) and *SOX10* negative regions. P-value after Mann-Whitney t-test. Data are represented as mean ± SD.
